# Spatiotemporal Dynamics of DENV-2 Asian-American Genotype Lineages in the Americas

**DOI:** 10.1371/journal.pone.0098519

**Published:** 2014-06-04

**Authors:** Daiana Mir, Hector Romero, Luiz Max Fagundes de Carvalho, Gonzalo Bello

**Affiliations:** 1 Laboratorio de Organización y Evolución del Genoma, Dpto. Ecología y Evolución, Facultad de Ciencias CURE, Universidad de la República, Montevideo, Uruguay; 2 Programa de Computação Científica - FIOCRUZ, Rio de Janeiro, Brazil; 3 Laboratorio de AIDS & Imunologia Molecular. Instituto Oswaldo Cruz - FIOCRUZ. Rio de Janeiro, Brazil; Singapore Immunology Network, Agency for Science, Technology and Research (A*STAR), Singapore

## Abstract

The Asian/American (AS/AM) genotype of dengue virus type 2 (DENV-2) has been evolving in the Americas over the last 30 years, leading to several waves of dengue epidemics and to the emergence of different viral lineages in the region. In this study, we investigate the spatiotemporal dissemination pattern of the DENV-2 lineages at a regional level. We applied phylogenetic and phylogeographic analytical methods to a comprehensive data set of 582 DENV-2 E gene sequences of the AS/AM genotype isolated from 29 different American countries over a period of 30 years (1983 to 2012). Our study reveals that genetic diversity of DENV-2 AS/AM genotype circulating in the Americas mainly resulted from one single founder event and can be organized in at least four major lineages (I to IV), which emerged in the Caribbean region at the early 1980s and then spread and die out with different dynamics. Lineages I and II dominate the epidemics in the Caribbean region during the 1980s and early 1990s, lineage III becomes the prevalent DENV-2 one in the Caribbean and South America during the 1990s, whereas lineage IV dominates the epidemics in South and Central America during the 2000s. Suriname and Guyana seem to represent important entry points for DENV-2 from the Lesser Antilles to South America, whereas Venezuela, Brazil and Nicaragua were pointed as the main secondary hubs of dissemination to other mainland countries. Our study also indicates that DENV-2 AS/AM genotype was disseminated within South America following two main routes. The first route hits Venezuela and the western side of the Andes, while the second route mainly hits Brazil and the eastern side of the Andes. The phenomenon of DENV-2 lineage replacement across successive epidemic outbreaks was a common characteristic in all American countries, although the timing of lineage replacements greatly vary across locations.

## Introduction

Dengue virus (DENV) is an archetypal emerging and re-emerging virus because it is an infection that appears as a new entity in certain areas where none has existed before and resurfaces in places where it already has existed [1]. There are four immunologically related single stranded RNA viruses that are classified as dengue serotypes (DENV-1 to 4). Each of the four DENV serotypes harbor extensive genetic diversity in the form of phylogenetically distinct clusters defined as genotypes and intra-genotype lineages that co-circulate with distinctive geographical and temporal patterns [2]. All these layers of viral diversity combined with host genetics and vector biology comprise the matrix responsible for the complex viral transmission dynamics of DENV at a global scale.

A common observation in phylogenetic studies of DENV is that of genotype replacement [3]. One of the best documented genotype replacement events occurred in the 1980s when the Asian/American (AS/AM) genotype of DENV-2 was introduced for the first time in the Americas, displacing the local American genotype [4]. Such replacement has been associated with a higher fitness of DENV-2 AS/AM genotype in both, humans and mosquitoes [5], [6], [7]. Since its introduction, estimated in the late 1970s [8], [9], the DENV-2 AS/AM genotype has been continuously circulating and evolving in the Americas leading to several waves of dengue hemorrhagic fever epidemics [10] and to the emergence of different viral lineages in the region. Phylogenetic analyses of the AS/AM genotype of DENV-2 in the Americas have identified the circulation of: three distinct clades in Puerto Rico (1986–2007) [11], [12] and the Lesser Antilles [13], two lineages in Bolivia (1997–2006) [14], Paraguay (2001–2006) [15] and Peru (1999–2012) [16], up to three lineages in Brazil (1990–2010) [17], [18], [19], [20] and Colombia (1988–2010) [21], up to six genetic clades in Venezuela (1991–2008) [22], [23], and two clades in Mexico (1996–2000) [24] and Central America (1999–2009) [25].

Information about the spatiotemporal dispersion dynamics of DENV-2 at regional level is scarce. A recent comprehensive study that analyzed 191 DENV-2 Asian–American genotype sequences isolated from different American countries between 1981 and 2008 suggests that this genotype was introduced to the Greater Antilles around 1979 and later spreads to the Lesser Antilles and northeastern coast of South America [8]. Dissemination within South America seems to have begun around 1992, and after 1995, Central America became involved as a result of the introduction from the islands. This study also indicates that gene flow between neighboring countries is more likely than between distant locales. Although this study provides an overall picture of the routes and the factors that influence the spread of DENV-2 AS/AM genotype in the Americas, there is no information about the spatiotemporal overlap and migration routes of the distinct genotype lineages previously described in the region.

The objective of this study was to reconstruct the origin and spatiotemporal dissemination dynamics of the major DENV-2 AS/AM lineages circulating in the Americas. To this end, we applied a model that combines DNA sequence data with its temporal and spatial information to a comprehensive data set of 582 DENV-2 E gene sequences of the AS/AM genotype isolated from 29 different American countries over a period of 30 years (1983 to 2012).

## Materials and Methods

### Sequence dataset

All complete (1,484 bp) DENV-2 and near complete (>1,400 bp) DENV-2 AS/AM genotype E gene sequences with known location and sampling date available in the GenBank by December 2012 were downloaded. This resulted in a final data set of 1,932 sequences from 63 countries isolated during 1944–2012 (Table 1). GenBank accession number, country of origin, and year of isolation of every included sequence is shown in Table S1. Nucleotide sequences were aligned using MAFFT v6.902b program [26]. Alignment is available from the authors upon request.

**Table 1:** Global DENV-2 data set.

Region	Country	*N*	Sampling dates
South America	Brazil	77	1990–2011
	Peru	40	2002–2012
	Venezuela	45	1990–2008
	Colombia	20	1944–2007
	Bolivia	12	1997–2010
	Suriname	11	1986–1999
	Others	10	1995–2010
Central America	Nicaragua	87	1999–2009
	Mexico	17	1992–2008
	Honduras	13	1984–2007
	Others	6	1987–2009
Caribbean	Puerto Rico	195	1977–2010
	Lesser Antilles	41	1981–2010
	Cuba	21	1997
	Other Greater Antilles	8	1983–2008
Asia	Singapore	475	2000–2011
	Vietnam	357	1988–2011
	Thailand	213	1964–2010
	Others	243	1956–2010
Others	-	51	1966–2011

Regions and countries within each region with the largest sampling size are indicated.

### Phylogenetic analysis

Sequence genotypes were confirmed through maximum likelihood (ML) tree estimation. A ML phylogenetic tree was inferred for the complete data set of 1,932 DENV-2 E gene sequences with the PhyML program [27], using an online web server [28]. Phylogenetic tree was reconstructed under the GTR+I + Г_4_ model of nucleotide substitution, selected using the jModelTest program [29]. Heuristic tree search was performed employing the SPR branch-swapping algorithm and the reliability of the phylogenies was estimated with the approximate likelihood-ratio test (a*LRT*) based on a Shimodaira–Hasegawa-like procedure.

### Analysis of spatiotemporal dispersion pattern

The rate of nucleotide substitution, the time to the most recent common ancestor (T_MRCA_) and the spatial diffusion of the DENV-2 AS/AM genotype in the Americas were jointly estimated, using Bayesian methods, with the Markov chain Monte Carlo (MCMC) algorithms implemented in the BEAST v1.7.5 package [30], [31]. In order to minimize the problem of over-representation of some countries, data sets were down-sampled to no more than 80 sequences per country. The temporal scale of evolutionary process was directly estimated from the sampling dates of the sequences using the GTR+I+ Г_4_ nucleotide substitution model, a relaxed uncorrelated lognormal molecular clock model [32], and a Bayesian Skyline coalescent tree prior [33]. Viral transmission pathways were reconstructed using a reversible discrete phylogeography model [34] and the rate of DENV-2 spread among locations was estimated using ‘Markov jump’ counts [35] of location-state transitions along the phylogeny as previously described [36], [37]. The MCMC analysis was run for 100 million generations and convergence of parameters was assessed by calculating the Effective Sample Size (ESS) using TRACER v1.5 [38] program, after excluding the initial 10% of the run. Uncertainty in parameter estimates was reflected in the 95% Highest Probability Density (HPD) values. The programs TreeAnnotator v1.7.5 and FigTree v1.4.0 [39] were used to summarize the posterior tree distribution and to visualize the annotated Maximum Clade Credibility (MCC) tree, respectively.

## Results

### Origin and dissemination of DENV-2 AS/AM genotype in the Americas

**Figure 1:**
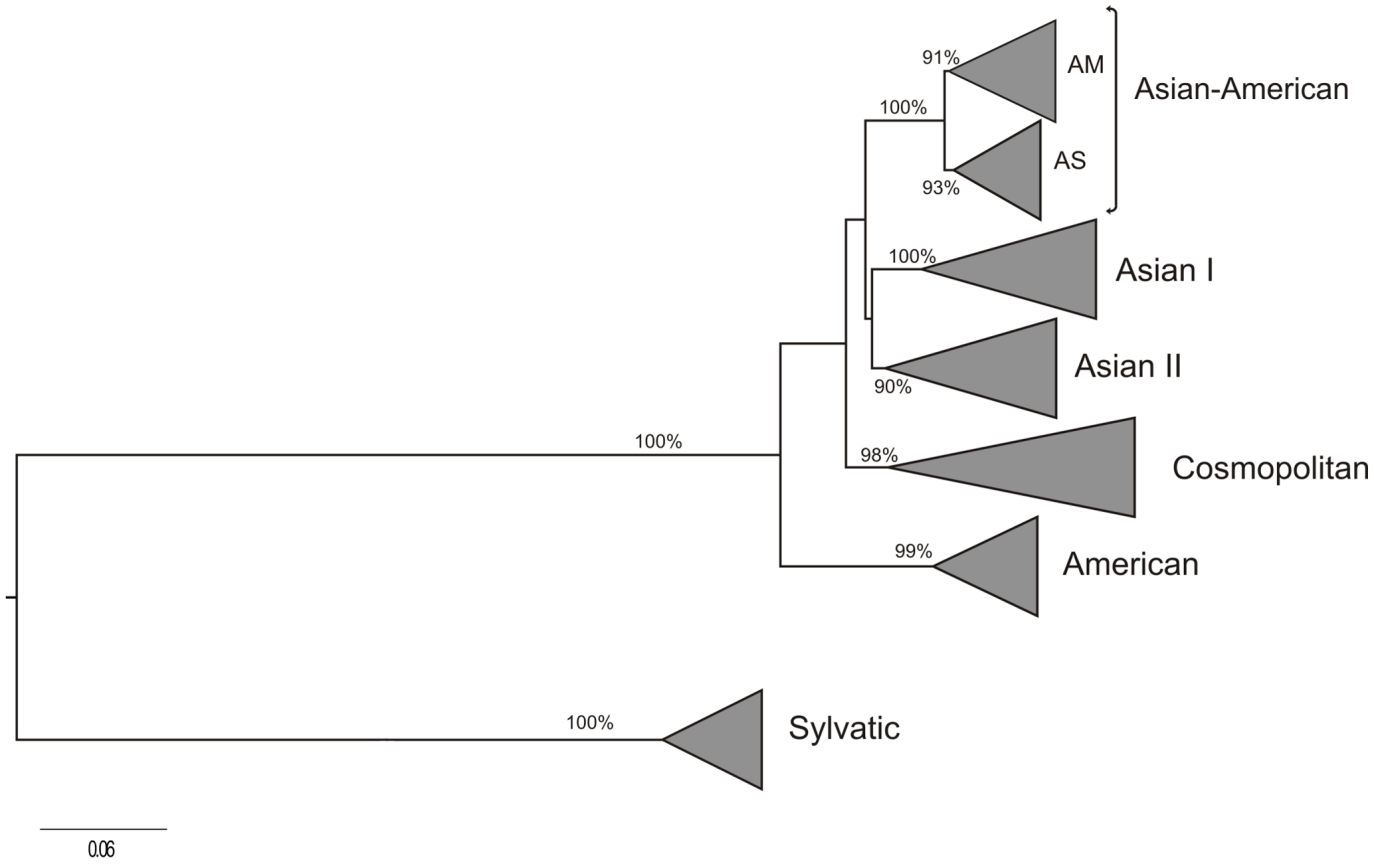
ML tree of 1,932 DENV-2 E gene sequences circulating globally. DENV-2 genotypes (Sylvatic, American, Asian I and II, Cosmopolitan and Asian/American) are identified. For visual clarity, strains from each genotype are shown collapsed. Only aLTR support values of major branching are shown. All horizontal branch lengths are drawn to a scale of nucleotide substitutions per site.

The ML tree of the complete data set of 1,932 DENV-2 E sequences, clearly shows the characteristic clustering of the non-sylvatic DENV-2 strains into five highly supported (a*LRT*≥0.90) monophyletic genotypes (Fig. 1). All DENV-2 AS/AM genotype sequences of American origin (*n* = 582) segregate in a highly supported (a*LRT* = 0.91) monophyletic sub-group nested within sequences of Asian origin, thus supporting that current diversity of DENV-2 AS/AM genotype sequences in the Americas mainly resulted from a single founder event. After excluding highly similar (>99.5%) sequences from the most densely sampled (*n*>80 sequences) locations (Puerto Rico and Nicaragua) and identical sequences from Cuba, we obtained a subset of 406 DENV-2 AS/AM genotype sequences of American origin that was used for subsequent evolutionary and phylogeographic reconstructions (Table 2).

**Table 2:** DENV-2 AS/AM genotype sequences of American origin.

Discrete geographic state^a^	Country	Complete dataset	Representative subset^b^	Sampling dates^c^
GA	Puerto Rico	192	79	1987–2010
	Cuba	21	1	1997
	Dominican Republic	3	3	1990–2003
	Jamaica	3	3	1983–2008
LA/SR/GY	Trinidad and Tobago	15	15	1986–2000
	Suriname	11	11	1986–1999
	Saint Vincent and the Grenadines	5	5	1998–2010
	Saint Lucia	4	4	1999
	Virgin Islands	4	4	1987–2005
	Barbados	3	3	1987–1998
	Dominica	3	3	1995
	Curacao	2	2	1993–1996
	Aruba	1	1	1996
	Grenada	1	1	1999
	Guyana	1	1	2000
	Saint Kitts and Nevis	1	1	2001
NI	Nicaragua	87	47	1999–2009
CAM/MX	Mexico	15	15	2002–2008
	Guatemala	2	2	2007–2009
	Honduras	2	2	2007
	Belize	1	1	2002
	Costa Rica	1	1	2003
VE	Venezuela	45	45	1990–2008
PE	Peru	38	38	2002–2012
CO/EC	Colombia	19	19	1992–2007
	Ecuador	4	4	2000
BR-SE	Brazil Southeastern region	59	59	1990–2011
BR-N/NE	Brazil Northern region	11	11	2000–2008
	Brazil Northeastern region	7	7	1991–2009
BO/PY	Bolivia	12	12	1997–2010
	Paraguay	6	6	2001–2010
TOTAL	-	582	406	1983–2012

a Neighboring countries with few sequences (*n*≤15) were merged in a single discrete geographic state. *^b^*Generated by removing highly similar (>99.5%) sequences from the most represented countries (Puerto Rico  = 113 and Nicaragua  = 40) and identical sequences from Cuba (*n* = 20). *^c^*Sampling dates of sequences were the same for both complete dataset and representative subset.

**Figure 2:**
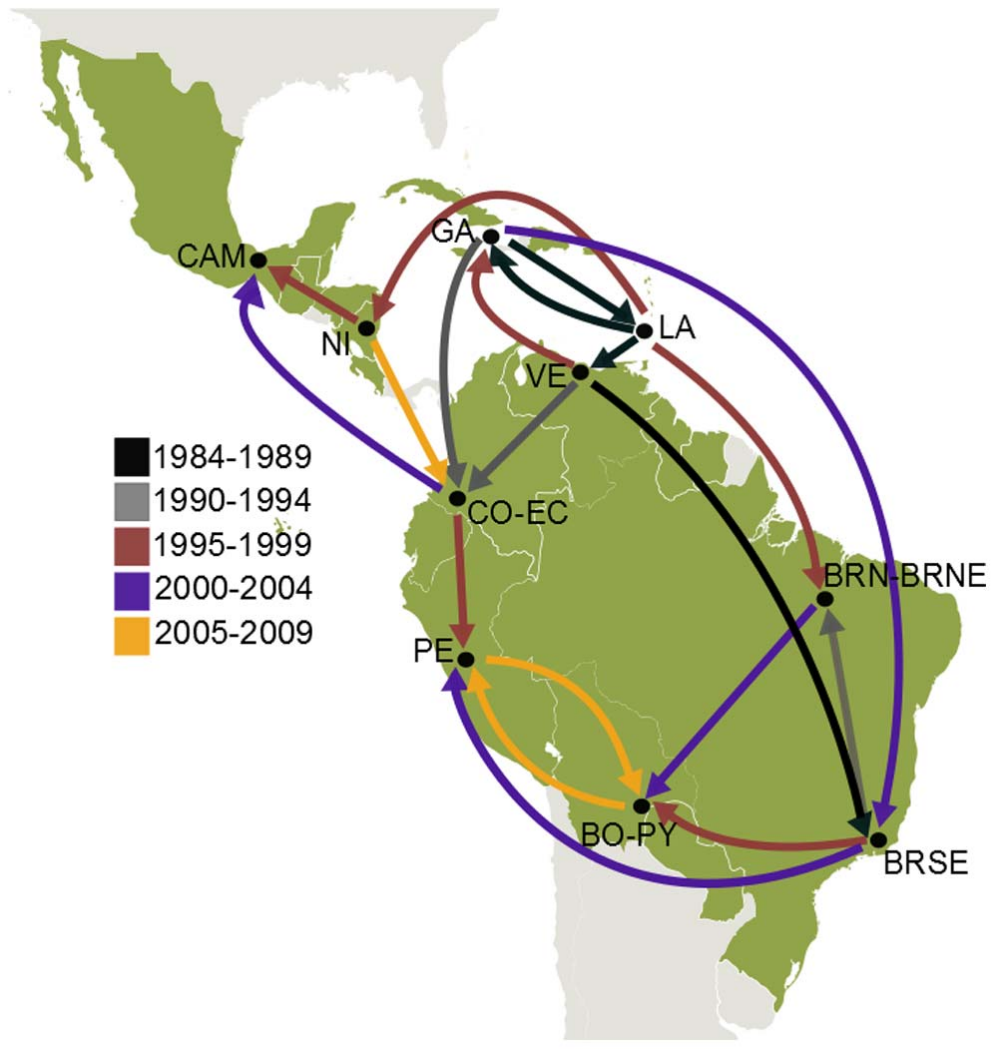
Spatiotemporal dynamics of dissemination of DENV-2 AS/AM genotype in the Americas. Viral dispersal pattern between 1984 and 2009. Lines between locations represent branches in the Bayesian MCC tree along which location transitions occurs. The color of lines informs the date of the earliest viral migrations among each pair of locations. (BO-PY: Bolivia–Paraguay, BRN–BRNE: Brazil Northern-Brazil Northeastern, BRSE: Brazil Southeastern, CAM: Central America-Mexico, CO-EC: Colombia-Ecuador, GA: Greater Antilles, LA: Lesser Antilles, NI: Nicaragua, PE: Peru, VE: Venezuela)

Ten discrete geographic states were initially assigned to DENV-2 American sequences (Table 2). The overall spatiotemporal dissemination pattern inferred for the DENV-2 AS/AM genotype in the Americas was fully consistent with that previously described by Allicock *et al* (2012) (Fig. 2). According to our estimations, the evolutionary rate of DENV-2 AS/AM genotype in the Americas was 9.5×10^−4^ subs./site/year (95% HPD: 8.5–10.7×10^−4^ subs./site/year) and the T_MRCA_ was the year 1981 (95% HPD: 1979–1983). Our analysis confirms that the DENV-2 AS/AM genotype was most probably introduced to the Greater Antilles (GA) (posterior state probability [*PSP*] = 0.69) and rapidly disseminated to the Lesser Antilles (LA) and the northeastern coast of South America (Suriname [SR] and Guyana [GY]). By the late 1980s the virus passed to Brazil and Venezuela and by the middle 1990s reached the other South American countries and entered into Central America from the Caribbean. Our phylogeographic analysis also reveals that viral dissemination within South America occurred following two major routes: the first one (western route) went through Venezuela (VE), Colombia (CO) and Ecuador (EC); whereas the second one (eastern route) began in Brazil (BR), continued to Bolivia (BO) and then Paraguay (PY) (Fig. 2). Both routes seem to finally converge in Peru (PE) that received viruses from both, western and eastern migration pathways.

**Figure 3:**
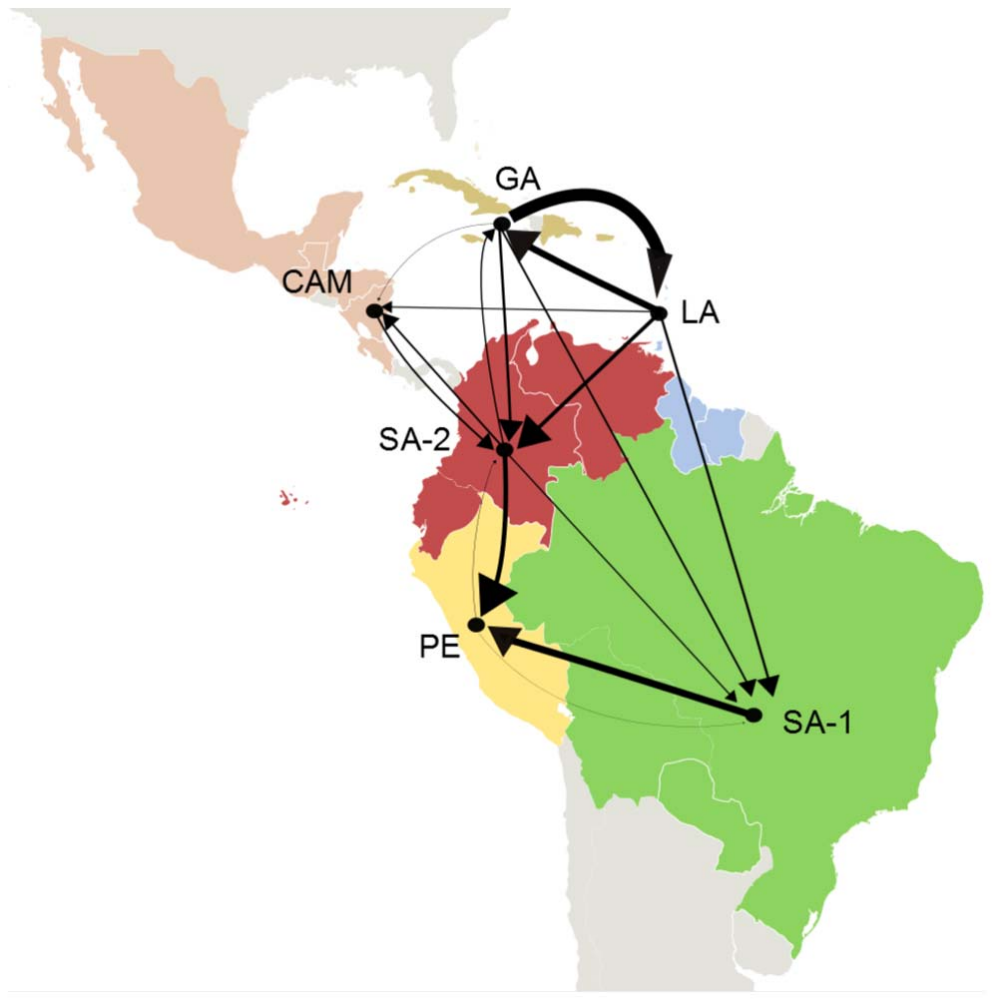
Major estimated viral migration rates among locations as measured using ‘Markov jump’ counts. The width of the arrows is proportional to the viral transitions rate. Transition rates lower than 0.1 were excluded for clarity. (CAM: Central America-Mexico, GA: Greater Antilles, LA: Lesser Antilles, PE: Peru, SA-1: South America 1, SA-2: South America 2)

In order to quantify the viral flux among regions and to reduce the potential impact of the unequal sampling size across locations, DENV-2 sequences were subdivided into six major locations with more homogenous sampling sizes named: GA (*n* = 86), LA/SR/GY (*n* = 51), SA_1_ (BR/BO/PY) (*n* = 95), SA_2_ (VE/CO/EC) (*n* = 68), PE (*n* = 38) and CAM (Central America and Mexico) (*n* = 68). Migration rates between those regions were estimated using Markov jump counts. The *PSP* distributions for the roots of major DENV-2 AS/AM lineages in the Americas obtained from the phylogeographic analyses with six and 10 geographic states were almost identical (Table S2). The highest viral transitions rates were: between GA and LA/SR/GY, from the Caribbean to both SA_1_ and SA_2_, from both SA_1_ and SA_2_ to PE and between SA_2_ and CEN (Fig. 3 and Table S3). The highest net viral migration rate (efflux minus influx) was for the Caribbean region (6.01), followed by SA_1_ (0.88), SA_2_ (0.40), CEN (−0.84) and PE (−6.45) regions.

### Diversification of DENV-2 AS/AM genotype in the Americas

**Figure 4:**
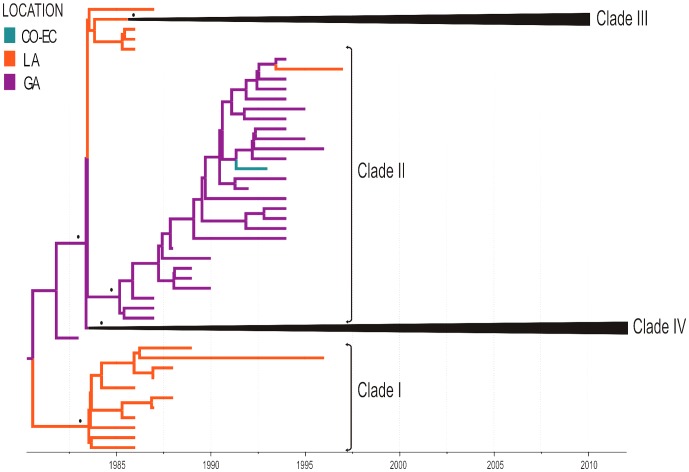
Time-scaled Bayesian Maximum Clade Credibility tree for the DENV-2 AS-AM genotype. Four major highly supported monophyletic clades (I-IV) are identified. Branches are colored according to the most probable location (legend shown on the left side) of their parental node inferred by discrete phylogeographical analysis. For visual clarity, clades III and IV has been collapsed (see Fig.5 and Fig.6). PP values >0.90 at key nodes are represented by (*). All horizontal branch lengths are drawn to a scale of years. The tree is automatically rooted under the assumption of a relaxed molecular clock. (CO-EC: Colombia-Ecuador, LA: Lesser Antilles, GA: Greater Antilles)

The evolutionary processes of DENV-2 AS/AM genotype in the Americas generated at least four major highly supported (posterior probability [*PP*] ≥0.95) lineages (I-IV) (Fig. 4). Lineages I and II were the most prevalent clades in the Caribbean region during the 1980s and early 1990s. Clade I probably emerged in the LA/SR (*PSP* = 1) in 1984 (95% HPD: 1983–1985) and only comprise older sequences from Barbados, Trinidad and Tobago and Suriname isolated during 1986–1996. Clade II probably arises in Puerto Rico (*PSP* = 1) in 1985 (95% HPD: 1984–1986) and was almost exclusively made up of sequences isolated in this island during 1987–1996. Lineages I and II appeared to become extinct around the middle 1996 and were replaced by lineages III and IV that were detected for the first time during the early 90s and have been responsible for the most dramatic DENV-2 epidemics in the Americas over the last 20 years.

### Time-scale and migration routes of DENV-2 AS/AM clade III

**Figure 5:**
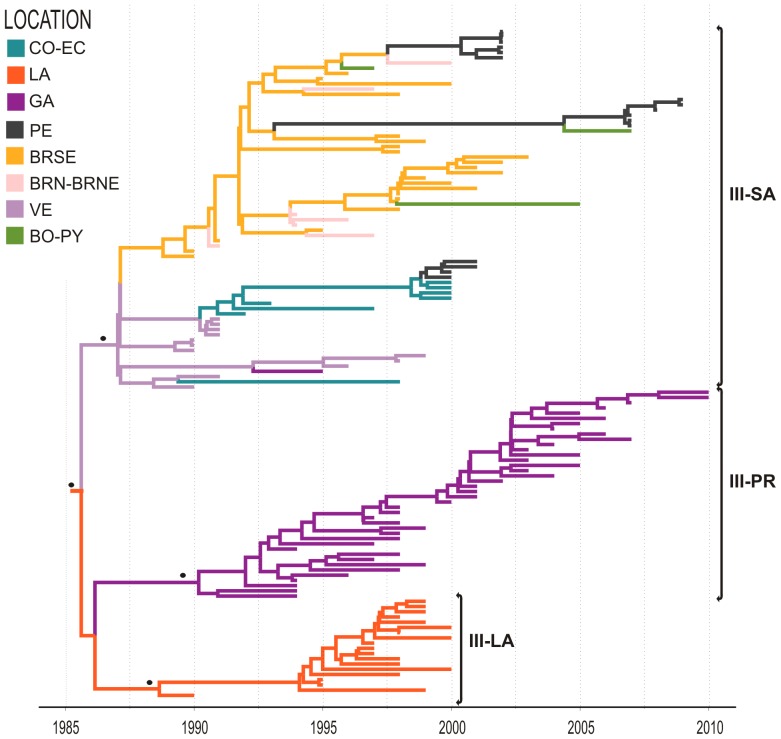
Time-scaled Bayesian Maximum Clade Credibility sub-tree corresponding to the Clade III. Three major highly supported monophyletic clades: III-PR, III-LA and III-SA are identified within clade III. For further details see legend of Fig 4. (CO-EC: Colombia-Ecuador, LA: Lesser Antilles, GA: Greater Antilles, PE: Peru, BRSE: Brazil Southeastern, BRN–BRNE: Brazil Northern-Brazil Northeastern, VE: Venezuela, BO-PY: Bolivia-Paraguay)

Clade III contains sequences from the LA, PR, and South America. This clade most likely emerged in the LA/SR/GY (*PSP* = 0.63) in 1986 (95% HPD: 1985–1987) and was disseminated throughout the Caribbean and South America, giving rise to three region-specific subclades (III-LA, III-PR, and III-SA) (Fig 5). The subclade III-LA emerged in 1988 (95% HPD: 1987–1990) and circulated in LA/GY between 1990 and 2000. The subclade III-PR arose in 1989 (95% HPD: 1988–1991) and only contains sequences isolated in PR between 1994 and 2010. This lineage co-circulated with clades II and IV-PR (see below) in Puerto Rico during the period of high prevalence of DENV-2 in 1994 and turned into the dominant viral clade in that country since mid 1990s [11], [12]. The subclade III-SA comprises South American sequences sampled between 1990 and 2009. This subclade probably originated in VE in 1987 (95% HPD: 1986–1988) and was disseminated in South America following two major concurrent routes. In the western route, the virus migrated from VE in sequential steps reaching CO in 1990 (95% HPD: 1990–1991), EC in 1998 (95% HPD: 1997–1999), and PE in 2000 (95% HPD: 1999–2001). In the eastern route, the virus entered the Southeastern Brazilian region in 1988 (95% HPD: 1986–1989) and was already detected in the North/Northeast Brazilian regions at 1991–1994. From BR the virus moved west being detected in BO in 1997, in PE in 2001 and in PY in 2005.

### Time-scale and migration routes of DENV-2 AS/AM clade IV

**Figure 6:**
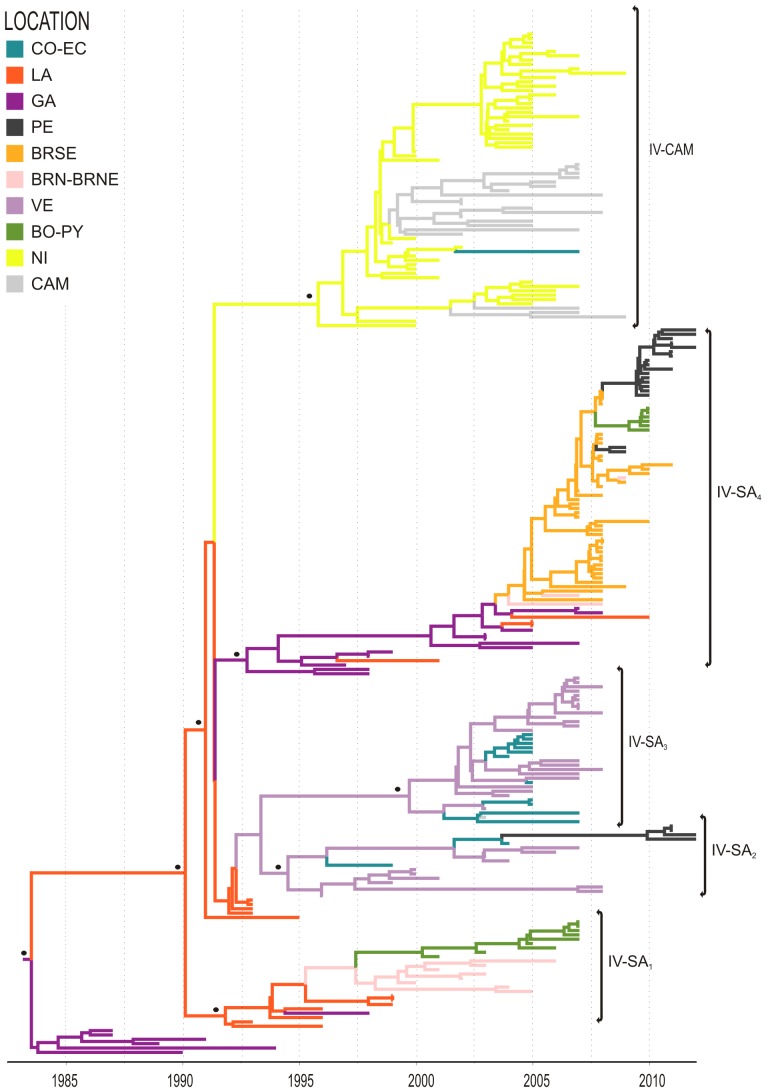
Time-scaled Bayesian Maximum Clade Credibility sub-tree corresponding to the Clade IV. Five major highly supported monophyletic clades: IV-SA_1–4_ and IV-CAM are identified within clade IV. For further details see legend of Fig 4. (CO-EC: Colombia-Ecuador, LA: Lesser Antilles, GA: Greater Antilles, PE: Peru, BRSE: Brazil Southeastern, BRN–BRNE: Brazil Northern-Brazil Northeastern, VE: Venezuela, BO-PY: Bolivia-Paraguay, NI: Nicaragua, CAM: Central America-Mexico)

Clade IV is the most widely disseminated DENV-2 lineage in the region and contains sequences from the Caribbean, South America and Central America. This clade most likely originated in PR (*PSP* = 0.95) in 1985 (95% HPD: 1984–1986) and was exported to the LA/SR in the same year (95% HPD: 1984–1986). From this location, the virus moved to South and Central America, giving rise to five regional subclades: four were disseminated within South America through the eastern (IV-SA_1_ and IV-SA_4_) and western (IV-SA_2_ and IV-SA_3_) migration routes and one was disseminated within Central America (IV-CAM) (Fig 6).

The subclade IV-SA_1_ comprises sequences isolated in SR during 1993–1999, in Northern Brazil during 2000–2006, in PY during 2001–2005, and in BO during 2003–2007. This subclade probably emerged in the LA (*PSP* = 1) around 1992 (95% HPD: 1991–1993), moved to Northern Brazil in 1999 (95% HPD: 1998–2000) and from BR to BO/PY in 2001 (95% HPD: 2000–2001). The subclade IV-SA_4_ contains sequences isolated in BR during 2007–2011, in BO and PY in 2010 and in PE during 2009–2012. This subclade probably arose with the introduction of a virus from the GA (*PSP* = 0.98) into the Southeastern Brazilian region (*PSP* = 0.55) in 2004 (95% HPD: 2003–2005). From this region, the virus moved to the Northeastern region as well as to BO, PY and PE.

The subclade IV-SA_2_ includes sequences isolated in VE during 1996–2008, in CO during 1999–2004, and in PE during 2011–2012. The origin of this subclade was traced back most probably to VE (*PSP* = 0.95) during 1995 (95% HPD: 1994–1996). From VE the virus was disseminated to CO and from CO the virus was later introduced into PE. The subclade IV-SA_3_ includes sequences isolated in VE during 2003–2008, in CO during 2004–2007, along with one sequence isolated in Costa Rica in 2003. This subclade most probably had an origin in VE (*PSP* = 0.78) in 1999 (95% HPD: 1997–2001) and from there the virus was disseminated to CO and then to Costa Rica.

Finally, the subclade IV-CAM contains sequences isolated in Belize, Guatemala, Honduras, Nicaragua (NI) and Mexico (MX) between 1999 and 2009, together with one sequence sampled in CO in 2007, being the first DENV-2 AS/AM lineage to become successfully established and disseminated in Central America. This subclade most probably was product of a migration from the LA (*PSP* = 0.98) into NI (*PSP* = 0.97) in 1997 (95% HPD: 1995–1998). NI contains the oldest sequences of subclade IV-CAM sampled in 1999–2000 and was probably the epicenter of the dissemination to other Central American countries, MX and CO, of this lineage. We found evidence of at least one successful introduction of this lineage into MX from NI (*PSP* = 0.93) around 1999 (95% HPD: 1998–2000).

## Discussion

The phylogeographic analysis of 582 DENV-2 AS/AM genotype sequences isolated from 29 American countries over a 30-year period confirms that regional viral diversity resulted from a single introduction of this genotype into the Americas around the early 1980s, followed by rapid spatial dispersion within the continent, in agreement with previous studies [8], [9]. Our phylogeographic analysis supports a primary spread axis between Greater and Lesser Antilles and secondary axes linking the Caribbean with both, South and Central America, also consistent with Allicock *et al* (2012). Caribbean islands have been also pointed as the initial source of DENV-1 and DENV-4 epidemics in the Americas [8], [40], [41] and as an important staging post in the dissemination of DENV-3 from Central to South America [42], [43], indicating that DENV migration events between the Caribbean and mainland regions are a frequent phenomenon in the Americas.

One important limitation of our study is the use of a highly biased dataset because sampled population only comprise a minor fraction of all DENV-2 cases occurred in the Americas over the last 30 years and sampling efforts respond to idyosincratic reasons including countries' specific public health policies and diverse research interests leading to important unbalances between countries [42]. Despite some limitations regarding both sampling procedures and model simplifications, this approach has proven to be rather robust for the study of DENV dissemination at regional level when data was interpreted cautiously within a sound biological framework [8], [9], [40], [41], [42], [43], [44], [45], [46], [47].

According to the results presented here, the Caribbean acted as a primary source of all DENV-2 lineages that were subsequently disseminated to continental regions; whereas Brazil, Venezuela and Nicaragua were pointed as the main secondary hubs of DENV-2 spreading to other mainland countries. Our results also point to a frequent intermix between sequences from the Lesser Antilles, Surinam, and Guyana, indicating that these latter countries represent important transit points for DENV-2 dissemination from the Caribbean to South America. The intense viral flux between the Lesser Antilles and Surinam/Guyana is probably facilitated by the close geographic proximity of those regions [8] as well as by linguistic and socioeconomic ties [9]. Thus, the phylogeographic pattern of DENV-2 in the Americas could be explained by a short-distance transmission model in which the virus moved outward from a central starting point located in the Caribbean to near continental regions which behave as secondary hubs of dissemination, transmitting the virus to other neighboring mainland countries. The backward movement of DENV-2 from mainland American countries to the Caribbean, by contrast, seems to be very reduced.

Our phylogeographic analysis also revealed the existence of two main routes of dissemination of DENV-2 AS/AM genotype within South America. The first route went to Venezuela and the western side of the Andes hitting Colombia, Ecuador and Peru, while the second route mainly hit the eastern side of the Andes, with Brazil passing the virus to Bolivia, Paraguay and finally Peru. Whereas viral exchanges between Venezuela/Colombia/Ecuador and Brazil/Bolivia/Paraguay seem to be very limited, Peru appears to be a confluence point of both dissemination routes, receiving viruses from Colombia/Ecuador through the northern border and from Brazil/Bolivia through the eastern one. A very similar pattern of viral spread was previously observed for DENV-3 in the Americas [42], [43], supporting the existence of two preferential corridors of DENV dissemination within South America common to all serotypes. These routes may be explained by the existence of the Andes Mountains that run north to south along the western coast of the continent and impose an important geographical barrier to both human and vector dissemination between South American countries located on both sides.

The genetic diversity of DENV-2 AS/AM genotype circulating in the Americas can be organized in four major genetically distinct lineages. Although all lineages arose in the same region (Caribbean) and around the same time (∼1985), they spread and die out with different dynamics across countries and regions. Lineages I and II arose in Lesser Antilles and Puerto Rico, respectively, and were mainly restricted to the Caribbean region where predominated until being replaced by clade III around the early 1990s. This latter clade probably emerged in the Lesser Antilles, became dominant in the Caribbean and South America during the 1990s and persisted as the prevalent lineage in Puerto Rico up to 2010. Clade IV probably arose in Puerto Rico and circulated in this country at low level until at least 2007; while became the dominant lineage in South and Central America from the early/middle 2000s onwards.

**Figure 7:**
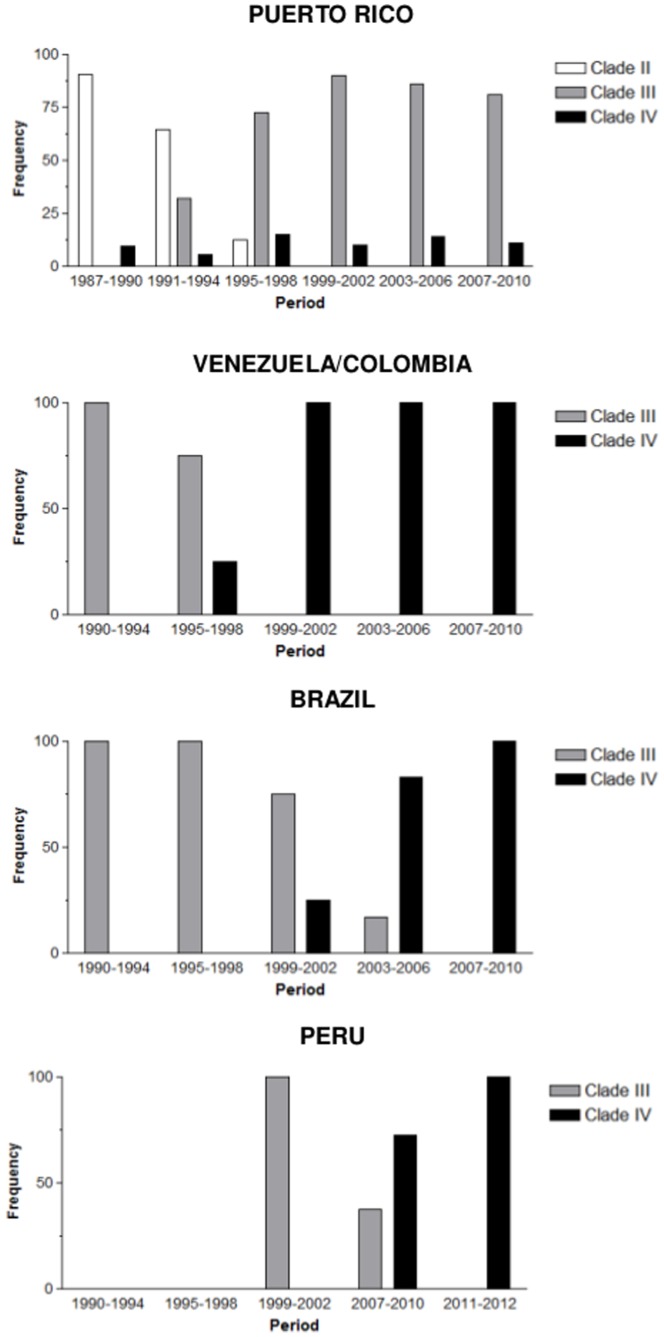
Distribution of DENV-2 AS/AM genotype sequences isolated in Puerto Rico (*n* = 195), Brazil (*n* = 77), Venezuela/Colombia (*n* = 64), and Peru (*n* = 38) at different time periods across the major phylogenetic lineages.

The phenomenon of DENV-2 lineage replacement across successive epidemic outbreaks was a common characteristic in all American countries; nevertheless, one fundamental difference was observed between Puerto Rico and the mainland regions (Fig. 7). DENV-2 evolution in the former was characterized by the long-term co-circulation of two or even three different AS/AM genotype lineages over time and this was particularly noticeable for clades III and IV that were simultaneously detected in Puerto Rico for nearly 15 years (1991–2007). The evolution of DENV-2 in South American countries, by contrast, seems to be characterized by short periods of overlap of distinct viral clades and more abrupt lineage replacements. We found evidence of co-circulation of clades III and IV for only six years in Venezuela (1993–1998), five years in Brazil (1999–2003), and one year in Peru (2009); while no evidence of co-circulation of different AS/AM genotype lineages was found in Central America.

Our study also suggests that timing of lineage replacements events greatly vary across South American countries. The dissemination process of DENV-2 clades III and IV in South America started in Venezuela and Brazil and took about 10 years to reach Peru by the western and eastern routes. Clade III probably arrived at Venezuela and Brazil around 1987–1989 and reached Peru in 2000–2001; whereas clade IV was probably introduced in Venezuela and Brazil around 1995–1997 and only hit Peru at 2006–2009. An important consequence of this rather slow dissemination process is that lineage replacement occurs in Peru much later than in the source countries (Venezuela and Brazil). In this same line of reasoning, Peru becomes an important sanctuary of DENV-2 lineages that become extinct in other South American countries. Clade III, for example, was detected in Peru until 2009, 6–10 years later than in Venezuela (1999) and Brazil (2003). It is interesting to note that Peru was also the last refuge of the DENV-2 American genotype in the Americas, being detected until the year 2000 [16].

One fundamental question is the relative contribution of stochastic processes and fitness differences among viral strains to DENV-2 lineage dissemination and turnover. Clade III has been established as the dominant DENV-2 lineage over the last 15 years in Puerto Rico, displacing clade II and prevailing over clade IV; but was replaced by clade IV in South America since the early 2000s onwards. Such variation across regions may be explained by population-specific stochastic process associated to temporal fluctuations in DENV serotype abundance as observed in Thailand [48]. In Brazil, clade III become extinct during the period of DENV-3 predominance (2000–2006), while clade IV was probably introduced around 2005 and fueled epidemics in the next period of high DENV-2 circulation (2007–2011) [20]. In Puerto Rico, by contrast, clade III was able to persist throughout the period of high DENV-3 circulation (1999–2003) by replicating in a refuge area and reemerged in the next season of high DENV-2 circulation [12]. Thus, population bottlenecks imposed by temporal fluctuations in DENV serotype abundance may be an important factor shaping within-genotype lineage extinctions and replacements, although their impact may vary across countries and time.

As we have mentioned before, the process of data collecting is an important limitation of our study and additional sampling among the locations and over time would allow more precise estimations of: 1) the prevalence, timing of introduction and temporal overlap of different viral clades across countries, and 2) the rate of viral exchanges among countries. Other findings of our study, however, are more robust to this limitation. First, the root location and dissemination routes of major DENV-2 American lineages inferred from different phylogeographic analyses were almost identical irrespective of the number and sampling size of locations assigned to South and Central American regions. Second, only estimates of rates of viral spread between major well sampled sub-regions were obtained, and not between countries with large differences in sampling sizes. Third, differences in DENV-2 lineage dynamics were detected across the most heavily sampled countries of our study (Puerto Rico, Brazil, Peru and Venezuela).

In summary, this study shows that the genetic diversity of DENV-2 AS/AM genotype circulating in the Americas over the last 30 years can be organized in at least four major genetically distinct lineages. The Caribbean islands together with Suriname and Guyana have been the primary source of DENV-2 lineages disseminated in the Americas; whereas Venezuela, Brazil and Nicaragua seem to be important hubs of viral dissemination to other mainland countries. Although all DENV-2 lineages emerged in the Caribbean region around the middle 1980s, they were disseminated and became extinct with very different dynamics. Continuous molecular surveillance of DENV epidemics in the Caribbean islands and northern coast of South America may contribute to identify new DENV-2 clades with potential to cause future epidemics in the region.

## Supporting Information

Table S1 **GenBank accession number, country of origin, and year of isolation of every sequence included in the analysis.**
(PDF)

Table S2 **Posterior state probability distributions for the roots of major DENV-2 American lineages estimated using two different schemes of grouping of discrete geographic states.**
(PDF)

Table S3 **Viral migration rates between locations.**
(PDF)
