# Supplementary material for: Spatiotemporal Dynamics of DENV-2 Asian-American Genotype Lineages in the Americas
Source: PLoS One. 2014 Jun 4;9(6):e98519. doi: 10.1371/journal.pone.0098519 (PMC4045713; doi:10.1371/journal.pone.0098519)
Supplement: Table S1 — GenBank accession number, country of origin, and year of isolation of every sequence included in the analysis. (PDF) [file pone.0098519.s001.pdf]

Table S1. Sequences

GB accession number	Country	Year of isolation
JF968045	TH	2010
EU448416	TH	2006
DQ181872	TH	2000
DQ181797	TH	2001
JQ815199	CN	2009
FJ744725	TH	2001
FJ744716	TH	2001
FJ744715	TH	2001
FJ744714	TH	2001
FJ687438	TH	2001
FJ687437	TH	2001
FJ639832	TH	2001
EU117352	TH	2001
EU117350	TH	2001
EU117341	TH	2001
EU117335	TH	2001
EU117330	TH	2001
EU117324	TH	2001
EU117318	TH	2001
JF967966	KH	2008
JF812111	TH	2006
JF812102	TH	2003
GU131924	KH	2008
GU131897	KH	2007
GQ868624	KH	2007
GQ868621	KH	2003
FJ898452	TH	2003
FJ639708	KH	2005
EU482661	VN	2006
DQ518646	TH	2004
JF968029	TH	2010
JF730045	KH	2008
GU211746	VN	2004
GU211745	VN	2004
GU131928	KH	2008
GU131900	KH	2008
FM210233	VN	2004
FJ744718	TH	2001
FJ744717	TH	2001
FJ639707	KH	2004
EU482775	VN	2004
EU117331	TH	2001
EU117326	TH	2001
EU117315	TH	2001
JF804027	VN	1988
GU131932	KH	2008
GU131901	KH	2008
GQ868625	KH	2008
GQ357789	SG	2008
FJ410221	VN	2007
EU482784	VN	2003
EU482782	VN	2003
EU482655	VN	2006
EU448415	KH	2007

JN556046	TH	2003
JQ403522	TW	2008
JN544398	SG	2011
JF968026	MM	2010
JF967990	TH	2009
JF967968	VN	2008
JF730046	KH	2008
GU908498	VN	2009
GU434158	VN	2004
GU434153	VN	2009
GU211739	VN	2004
GU131931	KH	2008
GU131902	KH	2008
GU131898	KH	2008
FM210240	VN	2004
FJ639718	KH	2008
FJ639704	KH	2003
FJ410215	VN	2008
EU482786	VN	2003
EU482677	VN	2006
EU482647	VN	2006
JF812112	TH	2006
JX093643	VN	2011
JF967987	KH	2009
JF967986	KH	2009
GU908510	VN	2009
FM210242	VN	2004
FM210231	VN	2004
EU482774	VN	2004
EU482642	VN	2006
EU482472	VN	2004
EU482468	VN	2006
EU482451	VN	2006
JF968033	TH	2010
JF968030	TH	2010
JF967989	KH	2009
JF967988	KH	2009
GU434148	VN	2007
GQ868543	TH	1995
FM210245	VN	2005
FM210216	VN	2004
FM210215	VN	2004
FM210211	VN	2003
FM210207	VN	2005
FJ906957	TH	1996
FJ410195	VN	2007
EU482776	VN	2005
EU482674	VN	2006
EU482665	VN	2006
EU482657	VN	2006
EU482470	VN	2006
EU482463	VN	2006
JX093646	VN	2011
JX093619	VN	2011
JX093605	VN	2011
JF968013	TH	2010

GU434147	VN	2007
GU211738	VN	2004
GQ868631	KH	2008
FM210246	VN	2005
FM210205	VN	2005
FJ744713	TH	2001
FJ687439	TH	2001
FJ639711	KH	2005
FJ639710	KH	2005
FJ410224	VN	2007
EU726776	VN	2007
EU687250	VN	2007
EU687248	VN	2007
EU660417	VN	2007
EU482675	VN	2006
EU482658	VN	2006
EU482447	VN	2006
EU117351	TH	2001
EU117339	TH	2001
DQ518645	TW	1998
DQ181866	TH	1996
JF968052	VN	2010
JN376791	VN	2008
JF812113	TH	2006
GU131886	TH	2001
FJ810412	TH	2001
FJ810411	TH	2001
FJ744724	TH	2001
FJ744712	TH	2001
FJ744711	TH	2001
FJ744710	TH	2001
FJ687447	TH	2001
FJ410208	VN	2007
FJ205877	VN	2007
FJ024461	VN	2007
FJ024458	VN	2007
FJ024452	VN	2007
EU687249	VN	2007
EU677149	VN	2007
EU660416	VN	2007
EU660415	VN	2007
EU482678	VN	2006
EU482663	VN	2006
EU482659	VN	2006
EU482649	VN	2006
EU482643	VN	2006
EU482448	VN	2006
EU482446	VN	2006
EU482445	VN	2006
EU448417	VN	2007
EU117345	TH	2001
EU117340	TH	2001
EU117334	TH	2001
EU117327	TH	2001
EU117325	TH	2001
EU117323	TH	2001

EU117317	TH	2001
DQ181900	TH	1997
DQ181849	TH	1995
JX101615	VN	2011
JX093617	VN	2011
JX093614	VN	2011
JN376793	VN	2010
JN376789	VN	2006
JF968021	LA	2010
JF968020	LA	2010
GU908505	VN	2009
GU908500	VN	2008
FM210206	VN	2005
FJ873811	VN	2006
FJ461311	VN	2008
FJ432724	VN	2007
FJ410288	VN	2007
FJ410233	VN	2008
FJ410217	VN	2007
FJ410200	VN	2007
FJ410193	VN	2007
EU660414	VN	2007
EU569721	VN	2006
EU482777	VN	2005
EU482703	VN	2007
EU482676	VN	2006
EU482653	VN	2006
EU482541	VN	2006
EU482466	VN	2006
EU482464	VN	2006
DQ518647	VN	2005
DQ181868	TH	1996
JX093638	VN	2011
JX093632	VN	2011
JX093600	VN	2011
JX093596	VN	2011
JX093595	VN	2011
JN376794	VN	2011
JF967984	VN	2009
HM134238	TH	1999
GU908499	VN	2008
GU131899	KH	2008
FJ461321	VN	2007
FJ432726	VN	2007
FJ410202	VN	2007
FJ390385	VN	2007
FJ373299	VN	2007
EU482705	VN	2007
EU482704	VN	2007
EU482669	VN	2006
EU482668	VN	2006
EU482656	VN	2006
EU482651	VN	2006
EU482646	VN	2006
EU482641	VN	2006
EU482543	VN	2006

EU482465	VN	2006
EU482450	VN	2006
DQ181871	TH	2001
DQ181841	TH	1991
JX093651	VN	2011
JX093633	VN	2011
JX093631	VN	2011
JX093630	VN	2011
JX093621	VN	2011
JX093615	VN	2011
JX093611	VN	2011
JX093603	VN	2011
JX093593	VN	2011
JX093591	VN	2011
GU908506	VN	2009
GU908503	VN	2008
GU908502	VN	2008
GU908501	VN	2008
GU434146	VN	2007
FJ410228	VN	2007
FJ410223	VN	2007
FJ390384	VN	2007
FJ205879	VN	2007
FJ205878	VN	2007
EU660413	VN	2007
EU482702	VN	2007
EU482699	VN	2007
EU482698	VN	2007
EU482697	VN	2007
EU482671	VN	2006
EU482652	VN	2006
EU482650	VN	2006
EU482474	VN	2007
EU482473	VN	2006
EU482467	VN	2006
DQ518648	VN	2005
JX093642	VN	2011
JX093641	VN	2011
JX093634	VN	2011
JX093623	VN	2011
JX093607	VN	2011
JQ896293	IE	2010
JN376790	VN	2007
JF968018	TH	2010
JF967993	VN	2009
GU908497	VN	2009
GU908494	VN	2009
GU434152	VN	2009
GU434149	VN	2008
FJ859028	VN	2007
FJ562098	VN	2007
FJ205880	VN	2007
EU677148	VN	2007
EU677138	VN	2007
EU677137	VN	2007
EU482701	VN	2007

EU482700	VN	2007
EU482679	VN	2007
EU482667	VN	2006
EU482644	VN	2006
EU482475	VN	2007
EU482471	VN	2006
DQ181894	TH	2000
JX093653	VN	2011
JX093652	VN	2011
JX093648	VN	2011
JX093645	VN	2011
JX093637	VN	2011
JX093629	VN	2011
JX093628	VN	2011
JX093625	VN	2011
JX093624	VN	2011
JX093620	VN	2011
JX093612	VN	2011
JX093608	VN	2011
JX093602	VN	2011
JX093599	VN	2011
JX093590	VN	2011
JX093589	VN	2011
JX093587	VN	2011
JQ403523	TW	2009
JF968041	TH	2010
JF968027	VN	2010
JF968019	VN	2010
JF968017	TH	2010
JF967978	VN	2009
GU908511	VN	2009
GU908508	VN	2009
GU908507	VN	2009
GU908495	VN	2009
GU131927	KH	2007
GQ868620	KH	2003
FJ547067	VN	2007
FJ547064	VN	2007
FJ410241	VN	2008
FJ410237	VN	2008
EU482673	VN	2006
EU482645	VN	2006
EU482469	VN	2006
JX093647	VN	2011
JX093644	VN	2011
JX093639	VN	2011
JX093636	VN	2011
JX093606	VN	2011
JX093601	VN	2011
JX093586	VN	2011
JF968048	VN	2010
JF967961	VN	2008
GU908504	VN	2008
GU434150	VN	2008
FJ461314	VN	2007
FJ024454	VN	2007

DQ181889	TH	2001
DQ181873	TH	2000
DQ181859	TH	1992
DQ181845	TH	1990
DQ181842	TH	1991
JX093649	VN	2011
JX093640	VN	2011
JX093635	VN	2011
JX093592	VN	2011
JX093588	VN	2011
JX093585	VN	2011
JF968042	TH	2010
JF967999	VN	2009
JF730049	VN	2007
FM210214	VN	2004
FJ687443	TH	2001
FJ687442	TH	2001
FJ687441	TH	2001
FJ687440	TH	2001
FJ461305	VN	2007
EU117329	TH	2001
EU117316	TH	2001
EU117314	TH	2001
EU117313	TH	2001
DQ181832	TH	1985
JX093626	VN	2011
JX093613	VN	2011
JX093610	VN	2011
JX093604	VN	2011
JX093598	VN	2011
FJ810409	TH	2001
FJ410259	VN	2008
EU482670	VN	2006
EU482654	VN	2006
EU482542	VN	2006
EU117349	TH	2001
DQ181844	TH	1990
JX093597	VN	2011
JN376792	VN	2009
FJ639706	KH	2004
DQ181848	TH	1987
DQ181843	TH	1990
DQ181829	TH	1987
JX093622	VN	2011
GQ868623	KH	2005
FJ639709	KH	2005
DQ518649	KH	2003
DQ181885	TH	1993
DQ181883	TH	1994
DQ181870	TH	1995
DQ181847	TH	1988
DQ181802	TH	1988
DQ518650	VN	2004
DQ181886	TH	1993
DQ181880	TH	1995
DQ181857	TH	1993

DQ181854	TH	1993
DQ181851	TH	1994
DQ181840	TH	1991
DQ181839	TH	1991
DQ181834	TH	1984
DQ181831	TH	1986
DQ181800	TH	1995
GQ868544	TH	1996
FJ461309	VN	2008
DQ181899	TH	1997
DQ181888	TH	1990
DQ181882	TH	1994
DQ181881	TH	1995
DQ181855	TH	1993
DQ181853	TH	1994
DQ181837	TH	1983
DQ181830	TH	1986
JF967994	VN	2009
GU908520	VN	2008
GU908518	VN	2008
GU908517	VN	2008
GU908516	VN	2008
GU908509	VN	2009
EU687246	TH	1994
DQ181901	TH	1996
DQ181897	TH	1998
DQ181835	TH	1984
DQ181803	TH	1985
JX093616	VN	2011
JN368476	KH	2007
GU908519	VN	2008
GU908513	VN	2008
GU908512	VN	2008
GU131896	KH	2007
GQ868638	KH	2008
FJ906958	TH	1996
FJ810410	TH	2001
FJ687446	TH	2001
FJ687445	TH	2001
FJ639829	TH	2001
FJ639717	KH	2007
FJ639705	KH	2003
EU117348	TH	2001
EU117322	TH	2001
EU117320	TH	2001
EU117319	TH	2001
DQ181891	TH	2000
DQ181852	TH	1994
DQ181850	TH	1994
JX093618	VN	2011
JX093654	VN	2011
JX093627	VN	2011
GU908515	VN	2008
GU908514	VN	2008
GQ868542	TH	1994
FJ744721	TH	2001

FJ687444	TH	2001
FJ639830	TH	2001
EU117347	TH	2001
EU117321	TH	2001
DQ181887	TH	1990
DQ181884	TH	1993
DQ181875	TH	1999
DQ181865	TH	1996
DQ181861	TH	1998
DQ181858	TH	1992
DQ181856	TH	1993
DQ181827	TH	1980
GQ868622	KH	2003
FJ744723	TH	2001
FJ744720	TH	2001
FJ744719	TH	2001
FJ639828	TH	2001
EU117346	TH	2001
EU117338	TH	2001
EU117337	TH	2001
EU117332	TH	2001
EU117328	TH	2001
DQ181893	TH	2000
DQ181892	TH	2000
DQ181876	TH	1999
DQ181867	TH	1996
JF730047	KH	2008
GU131930	KH	2008
DQ181879	TH	1996
DQ181869	TH	1995
DQ181860	TH	1998
JX093650	VN	2011
JF730048	KH	2008
FJ744722	TH	2001
FJ687436	TH	2001
FJ687435	TH	2001
FJ687434	TH	2001
EU117344	TH	2001
EU117343	TH	2001
EU117342	TH	2001
EU117336	TH	2001
DQ181878	TH	1998
DQ181864	TH	1997
DQ181863	TH	1997
GQ868545	TH	1996
FJ639831	TH	2001
EU117333	TH	2001
DQ518651	MM	2002
DQ181862	TH	1997
DQ181826	TH	1980
EU726767	TH	1994
DQ181896	TH	1999
DQ181828	TH	1980
DQ181821	TH	1982
DQ181805	TH	1979
DQ181895	TH	1999

DQ181833	TH	1985
DQ181809	TH	1979
DQ181817	TH	1983
DQ181798	TH	1999
DQ181890	TH	2001
DQ181877	TH	1999
DQ181823	TH	1981
DQ181804	TH	1984
DQ518652	MM	2004
DQ181874	TH	2000
DQ181824	TH	1981
DQ181819	TH	1982
DQ181838	TH	1983
FJ196851	CN	1998
DQ181822	TH	1982
JF812114	TH	2001
DQ518653	MM	2004
DQ181814	TH	1976
DQ181811	TH	1978
DQ181815	TH	1976
DQ181808	TH	1979
DQ181816	TH	1974
DQ181836	TH	1984
DQ181825	TH	1981
DQ181813	TH	1977
DQ181812	TH	1977
GU289914	TH	1974
DQ181806	TH	1974
DQ181807	TH	1980
DQ181810	TH	1978
GQ868591	TH	1964
GQ398268	ID	1975
JF730053	US	2006
JF730050	US	2007
JF730055	US	2009
JF730054	US	2009
HQ541798	US	2009
HQ541799	US	2010
HQ891023	TW	2008
EU854293	CO	1944
FJ390389	CO	1944
JN796245	CA	2008
JF804032	GU	2001
FJ906959	PG	2008
HQ891024	TW	2008
DQ181820	TH	1982
DQ181818	TH	1983
JN819418	VN	1988
GU211758	VN	2006
GQ398258	ID	1975
GU211754	VN	2006
GU211750	VN	2006
GQ398299	PR	1994
DQ518638	MM	1998
FJ538920	IN	1974
GQ398302	PR	1994

GQ398298	PR	1995
EU482747	PR	1988
EU482741	PR	1994
EU482585	PR	1989
EU482579	PR	1988
EU482578	PR	1988
EU482575	PR	1987
EU482569	PR	1987
GQ398300	PR	1994
GQ398264	ID	1976
JF804038	TT	1997
GU211763	VN	2006
GU211762	VN	2006
GU211760	VN	2006
GU211757	VN	2006
GU211752	VN	2006
GU211749	VN	2006
GU211747	VN	2006
GQ398307	PR	1994
GQ398306	PR	1994
GQ398303	PR	1994
GQ398301	PR	1995
EU660399	PR	1995
EU569705	PR	1993
EU569704	PR	1986
EU482743	PR	1989
EU482742	PR	1988
EU482737	PR	1994
EU482736	PR	1994
EU482588	PR	1990
EU482586	PR	1990
EU482582	PR	1989
EU482581	PR	1989
EU482577	PR	1987
EU482576	PR	1987
DQ181801	TH	1990
GQ398261	ID	1976
GQ398259	ID	1976
FJ538923	IN	1991
GU211761	VN	2006
GU211759	VN	2006
GU211756	VN	2006
GU211751	VN	2006
GQ398313	PR	1994
GQ398312	PR	1994
GQ398311	PR	1994
GQ398309	PR	1994
GQ398305	PR	1994
GQ398304	PR	1994
GQ398296	PR	1994
GQ398295	PR	1994
GQ398292	PR	1994
GQ398282	PR	1994
GQ398273	PR	1994
GQ398270	PR	1994
GQ398262	ID	1976

EU529700	PR	1994	
EU529694	PR	1994	
EU482746	PR	1989	
EU482744	PR	1989	
EU482739	PR	1994	
EU482584	PR	1989	
EU482583	PR	1989	
EU482574	PR	1987	
EU482573	PR	1987	
EU482570	PR	1987	
EU482568	PR	1986	
EU482562	PR	1989	
EU482561	PR	1996	
GQ398294	PR	1994	
FJ538922	IN	1983	
JN376785	VN	2002	
GU211755	VN	2006	
GQ868603	VI	1987	
GQ398310	PR	1994	
GQ398308	PR	1994	
GQ398297	PR	1994	
GQ398289	PR	1994	
GQ398283	PR	1994	
GQ398276	PR	1994	
GQ398275	PR	1994	
GQ398272	PR	1994	
EU660398	PR	1989	
EU482745	PR	1989	
EU482594	PR	1992	
EU482590	PR	1990	
EU482571	PR	1987	
GQ398263	ID	1975	
GQ398260	ID	1976	
GU434156	VN	2003	
GU434155	VN	2002	
GU434154	VN	2001	
GU211748	VN	2006	
GQ868541	VE	Aragua	1991
GQ398293	PR	1994	
GQ398291	PR	1994	
GQ398290	PR	1994	
GQ398288	PR	1994	
GQ398287	PR	1994	
GQ398286	PR	1994	
GQ398281	PR	1994	
GQ398280	PR	1994	
GQ398279	PR	1994	
GQ398274	PR	1994	
GQ398271	PR	1994	
FM210244	VN	2004	
EU569708	PR	1995	
EU569706	PR	1995	
EU529701	PR	1991	
EU482733	PR	1998	
EU482727	PR	1998	
DQ181846	TH	1988	

JX093594	VN	2011	
HM234642	BF	1986	
EU056810	BF	1983	
GU434159	VN	2004	
GQ398314	PR	1994	
GQ398285	PR	1994	
GQ398278	PR	1994	
GQ398277	PR	1994	
FM210243	VN	2004	
FM210238	VN	2001	
FM210226	VN	2002	
FJ898450	VI	1990	
FJ538911	IN	1993	
EU569707	PR	1995	
EU529695	PR	1994	
EU482740	PR	1994	
EU482738	PR	1998	
EU482721	PR	2006	
EU482589	PR	1993	
EU482587	PR	1990	
EU482580	PR	1989	
AM746224	SA	1994	
AM746223	SA	1994	
AB479042	VN	2002	
AB479041	VN	2002	
JF968039	PH	2010	
JF957000	CN	2010	
EF016253	TW	2002	
JN376787	VN	2004	
GQ868552	CO	Santander	1998
FM210228	VN	2003	
FM210217	VN	1999	
FM210202	VN	2004	
FJ538925	IN	1992	
EU660400	PR	1998	
EU596487	PR	1998	
EU569717	PR	1997	
EU569715	PR	1997	
EU569712	PR	1998	
EU569709	PR	1996	
EU569703	PR	1996	
EU482735	PR	1998	
EU482734	PR	1998	
EU482729	PR	1999	
EU482572	PR	1987	
EU482550	PR	1998	
DQ518641	VN	2003	
DQ518630	TW	2001	
GQ398269	PR	1994	
JF968001	PH	2009	
GU131843	BF	1986	
EF016252	TW	2002	
EF016251	TW	2002	
EF016250	TW	2002	
GU211753	VN	2006	
GQ868598	VE	1991	

GQ868597	VE	1991	
GQ868596	VE	1991	
GQ868595	VE	1991	
GQ868540	VE	Maracay	1990
GQ398284	PR	1994	
FM210234	VN	2004	
FM210230	VN	2003	
FM210218	VN	2002	
FJ639697	KH	2001	
EU596485	PR	1996	
EU569716	PR	1997	
EU569714	PR	1997	
EU569711	PR	1998	
EU529706	PR	2006	
EU482732	PR	1998	
EU482664	VN	2006	
EU482565	PR	1998	
AM746227	SA	1994	
AM746225	SA	1994	
JN851131	SG	2005	
JN819416	NI	2000	
JF967955	PH	2008	
GQ199898	NI		2001
GQ199895	NI	1999	
GQ199894	MX	2005	
FJ873808	NI	2000	
FJ850060	NI	2000	
EU482634	NI	2006	
DQ518631	PH	2005	
JF730044	KH	2001	
GQ868553	CO	Santander	1999
FM210229	VN	2003	
FM210223	VN	2003	
FM210219	VN	2003	
FM210212	VN	2003	
FM210208	VN	2003	
FM210203	VN	2003	
FJ898465	VE	Aragua	1998
EU726775	VE	Aragua	1996
EU687225	PR	2000	
EU687222	PR	2000	
EU687220	VE	Aragua	1996
EU677141	PR	1996	
EU596486	PR	1996	
EU569719	PR	1997	
EU569718	PR	1997	
EU569710	PR	1996	
EU482779	VN	2003	
EU482778	VN	2003	
EU482730	PR	1999	
EU482728	PR	1998	
EU482557	PR	1999	
EU482545	PR	1998	
L10045	PH	1983	
JF968037	PH	2010	
JF967971	PH	2008	

GQ868497	MX	2006	
FJ850119	NI	2000	
FJ850118	NI	2000	
FJ850117	NI	2000	
FJ850054	NI	2005	
FJ226066	NI	2005	
EU482761	NI	2005	
EU482754	NI	2005	
EU482748	NI	2005	
EU482695	NI	2005	
EU482681	NI	2005	
EU482680	NI	2005	
EU482631	NI	2005	
EU482602	NI	2005	
EU482599	NI	2005	
EU482598	NI	2005	
JF967956	ID	2008	
FM210239	VN	2004	
FM210232	VN	2004	
FM210225	VN	2001	
FM210224	VN	2001	
FM210222	VN	2004	
FM210220	VN	2003	
FM210213	VN	2005	
FM210210	VN	2003	
FM210204	VN	2003	
FJ898460	KN	2001	
FJ882602	LK	1996	
FJ639698	KH	2002	
FJ538912	IN	1995	
FJ024477	CO	Antioquia	2004
EU687232	PR	2001	
EU687227	PR	2001	
EU687224	PR	2000	
EU687223	PR	2000	
EU687215	PR	2004	
EU687213	PR	2004	
EU677144	PR	1999	
EU677143	PR	1999	
EU569720	PR	1997	
EU569713	PR	1997	
EU529693	PR	2004	
EU482788	VN	2003	
EU482783	VN	2003	
EU482781	VN	2003	
EU482722	PR	2002	
EU482554	PR	1998	
EU482547	PR	1998	
DQ518640	TW	2005	
JN030343	SG	2010	
JF968047	PH	2010	
JF968036	PH	2010	
JF968011	PH	2010	
JF804029	CO	2007	
GU586492	GT	2007	
GQ199897	NI	2002	

GQ199893	MX	2002	
GQ199874	NI	2004	
FJ906961	NI	2005	
FJ898478	NI	2000	
FJ898436	NI	2004	
FJ882594	NI	2007	
FJ850066	NI	2000	
FJ850063	NI	2000	
FJ850062	NI	2000	
FJ850053	NI	2005	
FJ744705	NI	2000	
FJ196852	CN	2001	
EU482757	NI	2005	
EU482755	NI	2005	
EU482689	NI	2006	
EU482444	NI	2006	
EF051521	CN	2001	
JN030344	SG	2010	
GU211744	VN	2003	
FJ639788	VE	Aragua	2004
FJ639783	VE	Aragua	2003
FJ639703	KH	2003	
FJ639701	KH	2002	
FJ639699	KH	2002	
FJ538924	IN	1990	
EU726770	PR	2005	
EU687244	PR	2004	
EU687243	PR	2004	
EU687242	PR	2004	
EU687240	PR	2003	
EU687237	PR	2003	
EU687231	PR	2001	
EU687229	PR	2001	
EU687228	PR	2001	
EU687212	PR	1998	
EU687199	PR	2003	
EU677147	PR	2004	
EU482787	VN	2003	
EU482785	VN	2003	
EU482780	VN	2003	
EU482724	PR	2005	
EU448421	IN	2006	
JN380838	SG	2009	
JF967998	PH	2009	
JF967997	PH	2009	
JF967995	PH	2009	
JF967991	PH	2009	
JF967983	PH	2009	
JF327392	SG	2009	
HQ541793	NI	2005	
HM631868	NI	2005	
FM986656	MY	1999	
FJ937969	MX	2005	
FJ937968	MX	2005	
FJ906956	NI	2005	
FJ898477	NI	2000	

FJ850121	NI	2001	
FJ850120	NI	2001	
FJ850067	NI	2006	
FJ850065	NI	2000	
FJ850064	NI	2000	
FJ850061	NI		2000
FJ744741	NI	2006	
EU482771	NI	2005	
EU482763	NI	2005	
EU482760	NI	2005	
EU482759	NI	2005	
EU482756	NI	2005	
EU482752	NI	2005	
EU482639	NI	2006	
EU482629	NI	2007	
EU482603	NI	2007	
EF540856	TW	1981	
DQ518632	PH	2001	
AM746226	SA	1994	
JN819408	VE	Aragua	2001
JF967972	ID	2008	
JF804031	DO	2003	
GU434157	VN	2004	
GU211742	VN	2003	
GU211741	VN	2003	
GU211740	VN	2004	
FM210237	VN	2003	
FM210235	VN	2005	
FM210227	VN	2002	
FM210209	VN	2003	
FJ898451	DO	2003	
FJ639702	KH	2003	
EU687241	PR	2003	
EU687238	PR	2003	
EU687236	PR	2003	
EU687235	PR	2003	
EU687230	PR	2001	
EU687214	PR	2004	
EU596489	PR	2007	
EU482731	PR	2005	
EU482725	PR	2006	
EU482560	PR	1998	
EU482553	PR	2006	
EU482544	PR	2006	
EU448419	KH	2004	
JF968005	MY	2010	
JF804035	MX	2002	
HQ541801	NI	2005	
HQ541794	NI	2005	
GU131975	MX	2007	
GU131959	MX	2006	
GU131955	MX	2004	
FM986661	MY	2002	
FM986658	MY	2002	
FJ898438	MX	2002	
FJ898435	NI	2005	

FJ850115	NI	2005	
EU596484	NI	2005	
EU482770	NI	2005	
EU482769	NI	2005	
EU482758	NI	2005	
EU482753	NI	2005	
EU482750	NI	2005	
EU482749	NI	2005	
EU482696	NI	2006	
EU482637	NI	2005	
EU482636	NI	2005	
EU482635	NI	2005	
EU482633	NI	2006	
EU482632	NI	2005	
EU482600	NI	2005	
EU482597	NI	2005	
JN376786	VN	2003	
JF968040	IN	2010	
JF967973	ID	2009	
JF967969	ID	2008	
JF804037	VI	2005	
FM210236	VN	2004	
FJ898466	VE	Aragua	2000
FJ898453	VI	2005	
FJ639822	VE	Aragua	2006
FJ639700	KH	2002	
EU677145	PR	1999	
EU677142	PR	1999	
EU482593	PR	2001	
EU045311	PY	2001	
DQ448232	IN	2001	
JX079692	NI	2007	
JN819422	MX	2008	
JF968038	PH	2010	
JF968004	PH	2010	
HQ541788	NI	2007	
GU131974	MX	2007	
GQ868646	NI	2007	
GQ868516	MX	2007	
FM986657	MY	2002	
FJ906962	NI	2005	
FJ898439	MX	2008	
FJ744704	NI	2001	
FJ410291	NI		2007
FJ390390	NI	2007	
EU569699	NI	2007	
EU482762	NI	2005	
EU482672	VN	2006	
DQ518635	MY	2003	
JF968008	ID	2010	
JF804036	PR	2007	
GU211743	VN	2003	
GQ252676	LK	2003	
FM210241	VN	2004	
FM210221	VN	2003	
EU781135	PR	2005	

EU687245	PR	2005
EU687217	PR	2005
EU687216	PR	2005
EU677146	PR	2004
EU596491	PR	2007
EU596490	PR	2007
EU596488	PR	2007
EU482726	PR	2006
EU482723	PR	2002
EU482551	PR	2006
EU482548	PR	2006
EU482546	PR	1998
EU448431	ID	2006
DQ518642	VN	2004
DQ448236	IN	2001
AM746222	SA	2004
AM746221	SA	2004
JN030325	SG	2010
JF968046	PH	2010
JF967967	ID	2008
HM488257	GU	2001
GQ357793	SG	2008
FJ898461	BZ	2002
FJ196853	CN	2003
EU482688	NI	2006
EU482640	VN	2006
EU448425	ID	2007
EU179858	BN	2005
EU179857	BN	2005
DQ518643	PH	1994
DQ518636	MY	2004
DQ518633	PH	2005
JX402215	PR	2010
JX402214	PR	2010
JQ013409	PR	2007
JQ013408	PR	2007
JN544396	SG	2011
JN544395	SG	2011
JN544394	SG	2011
JN376788	VN	2005
JF968022	ID	2010
JF967963	ID	2008
EU482720	PR	2006
EU482719	PR	2006
EU482556	PR	2005
EU482552	PR	2006
EU482549	PR	2006
DQ448233	IN	2001
DQ448231	IN	2001
JX093609	VN	2011
JQ403524	TW	2010
JN544388	SG	2011
JN380861	SG	2010
JN196612	SG	2009
JF968015	ID	2010
JF968006	ID	2010

JF967996	PH	2009	
JF967977	ID	2009	
JF967970	ID	2008	
HQ999999	GT	2009	
HQ705624	NI	2009	
HQ541805	NI	2005	
GU586123	HN	2007	
GQ868515	MX	2007	
EU448426	PH	2007	
EU069587	SG	2003	
EU069581	SG	2005	
JN030345	SG	2010	
JF968007	ID	2010	
JF804034	JM	2008	
GQ252677	LK	2004	
GQ199892	JM	2007	
FM986654	MY	1997	
FJ850107	VE	Aragua	2008
FJ538913	IN	1997	
FJ538905	IN	2004	
EU482666	VN	2006	
EU045312	PY	2005	
JQ896295	IE	2011	
JN544393	SG	2011	
JN544392	SG	2011	
JN544387	SG	2011	
JN544386	SG	2011	
JN544385	SG	2011	
JN544384	SG	2011	
JN544383	SG	2011	
JN544382	SG	2011	
JN380860	SG	2010	
JN030326	SG	2010	
JF968016	ID	2010	
JF967964	ID	2008	
GU586122	HN	2007	
GQ357795	SG	2003	
EU179859	BN	2006	
EU069576	SG	2005	
JF968043	VC	2010	
FJ898467	VE	Aragua	2005
JN851126	SG	2005	
JN851125	SG	2005	
JN851124	SG	2005	
JN544399	SG	2011	
JN544390	SG	2011	
JN544389	SG	2011	
JN196611	SG	2009	
JN036376	BD	2008	
JF968024	ID	2010	
JF968014	ID	2010	
JF968012	MY	2010	
GU908496	VN	2009	
GQ357796	SG	2003	
GQ357788	SG	2000	
GQ357783	SG	2005	

GQ357776	SG	2001	
GQ357775	SG	2004	
GQ357773	SG	2004	
GQ357771	SG	2003	
GQ357770	SG	2003	
FM986659	MY	2002	
FJ196854	CN	1993	
EU448422	IN	2004	
EU448418	PH	2003	
EU081179	SG	2005	
EU081178	SG	2005	
EU081177	SG	2005	
EU069589	SG	2003	
EU069588	SG	2003	
EU069586	SG	2003	
EU069583	SG	2000	
EU069579	SG	2004	
DQ518637	ID	2005	
JN036379	BD	2006	
JN036378	BD	2007	
JN036374	BD	2009	
JN036372	BD	2009	
JN030333	SG	2010	
JN030328	SG	2010	
JF804033	IN	2006	
HQ332187	VE	2007	
HQ332185	VE	2007	
GQ868554	CO	Santander	2004
FJ898454	IN	2006	
FJ807640	IN	1994	
FJ639734	VE	Aragua	2003
FJ639732	VE	Aragua	2005
EU482662	VN	2006	
EU482648	VN	2006	
EU482607	VE	Aragua	2007
EU448423	BD	2005	
EU448420	VN	2006	
DQ518639	VN	2005	
JN851130	SG	2005	
JN851128	SG	2006	
JN544391	SG	2011	
JN380816	SG	2009	
JN196568	SG	2009	
JN030327	SG	2009	
JF968035	MY	2010	
JF968002	ID	2009	
JF967976	ID	2009	
JF967975	ID	2009	
GQ357794	SG	2003	
GQ357790	SG	2007	
GQ357787	SG	2003	
GQ357785	SG	2003	
FM986660	MY	2002	
EU448430	ID	2007	
EU069592	SG	2003	
EU069584	SG	2002	

EU069580	SG	2003	
EU069577	SG	2004	
EU069575	SG	2003	
EU069573	SG	2003	
EU069572	SG	2002	
JN030334	SG	2010	
JN030331	SG	2010	
JN030330	SG	2010	
JN030329	SG	2010	
HQ332190	VE	2007	
HQ332188	VE	2007	
HQ332184	VE	2006	
GQ868557	CO	Santander	2005
GQ868556	CO	Santander	2005
GQ868555	CO	Santander	2005
FJ850108	VE	Aragua	2008
FJ850106	VE	Aragua	2008
EU482660	VN	2006	
EU482608	VE	Aragua	2007
EU482606	VE	Aragua	2007
EU482604	VE	Aragua	2007
EU045313	PY	2005	
DQ448237	IN	2001	
DQ448234	IN	2001	
JN851123	SG	2004	
JN036373	BD	2009	
JN030207	SG	2008	
JN030200	SG	2008	
JN030199	SG	2008	
JN022603	SG	2008	
JF968023	ID	2010	
JF968009	ID	2010	
JF968003	ID	2010	
JF968000	ID	2009	
GU370051	SG	2008	
GQ357792	SG	2002	
GQ357791	SG	2007	
GQ357784	SG	2005	
GQ357781	SG	2008	
GQ357774	SG	2005	
GQ357768	SG	2008	
GQ357767	SG	2008	
GQ357714	SG	2008	
EU448427	SG	2007	
EU081180	SG	2005	
EU069574	SG	2005	
JN544397	SG	2010	
JN196614	SG	2010	
JN196613	SG	2010	
JN036377	BD	2008	
JN030340	SG	2010	
HQ332189	VE	2007	
HQ332186	VE	2007	
FJ850112	VE	Caracas	2004
FJ639733	VE	Aragua	2005
EU482605	VE	Aragua	2007

EU482449	VN	2006	
EU448424	BD	2004	
DQ448235	IN	2001	
JN851122	SG	2008	
JN030238	SG	2008	
JN030232	SG	2008	
JN030210	SG	2008	
JN030209	SG	2008	
JN030208	SG	2008	
JF967962	MY	2008	
GQ357824	SG	2008	
GQ357819	SG	2008	
GQ357816	SG	2008	
GQ357798	SG	2004	
GQ357797	SG	2004	
GQ357786	SG	2004	
GQ357764	SG	2008	
GQ357762	SG	2008	
GQ357759	SG	2008	
GQ357756	SG	2008	
GQ357724	SG	2008	
GQ357723	SG	2008	
GQ357721	SG	2008	
GQ357720	SG	2008	
GQ357718	SG	2008	
EU069591	SG	2004	
EU069590	SG	2004	
AB545874	JP	2008	
JN036380	BD	2006	
JN036375	BD	2008	
JF804030	CR	2003	
GU131947	CO	Santander	2007
GQ868558	CO	Santander	2007
FJ850105	VE	Aragua	2007
FJ538906	IN	2005	
JN851119	SG	2008	
JN851114	SG	2007	
JN851113	SG	2006	
JN380844	SG	2009	
JN196610	SG	2009	
JN196579	SG	2009	
JN196578	SG	2009	
JN196577	SG	2009	
JN196576	SG	2009	
JN196575	SG	2009	
JN196574	SG	2009	
JN030317	SG	2007	
JN030271	SG	2007	
JF968028	MY	2010	
JF968010	ID	2010	
JF967974	ID	2009	
GQ398267	SG	2007	
GQ398266	SG	2007	
GQ398265	SG	2008	
GQ357828	SG	2007	
GQ357810	SG	2008	

GQ357804	SG	2007	
GQ357800	SG	2007	
GQ357799	SG	2007	
GQ357782	SG	2007	
GQ357717	SG	2008	
GQ357711	SG	2008	
EU448429	SG	2007	
JN819407	VE	Merida	2007
JN030339	SG	2010	
JN030338	SG	2010	
JN030337	SG	2010	
JN030336	SG	2010	
JN030335	SG	2010	
JN030332	SG	2010	
JF967992	IN	2009	
GQ868641	VE	Aragua	2007
EU854294	CO	Guaviare	2005
JN851117	SG	2007	
JN851120	SG	2007	
JN851116	SG	2007	
JN380841	SG	2009	
JN380830	SG	2009	
JN030274	SG	2007	
JN030273	SG	2007	
JN030247	SG	2009	
JN030234	SG	2010	
JN030216	SG	2008	
JN030213	SG	2009	
JN030206	SG	2008	
JN030201	SG	2008	
JN022602	SG	2007	
JN009091	CN	2010	
JF968050	ID	2010	
JF968031	ID	2010	
JF967981	ID	2009	
JF967960	MY	2008	
JF967954	ID	2008	
GU370050	SG	2007	
GQ357829	SG	2007	
GQ357825	SG	2007	
GQ357778	SG	2007	
GQ357763	SG	2008	
GQ357754	SG	2008	
GQ357751	SG	2008	
GQ357750	SG	2008	
GQ357749	SG	2008	
GQ357748	SG	2008	
GQ357747	SG	2008	
GQ357746	SG	2008	
GQ357744	SG	2008	
GQ357743	SG	2008	
GQ357742	SG	2008	
GQ357741	SG	2008	
GQ357740	SG	2008	
GQ357739	SG	2007	
GQ357738	SG	2007	

GQ357737	SG	2007	
GQ357736	SG	2006	
GQ357735	SG	2007	
GQ357734	SG	2007	
GQ357733	SG	2007	
GQ357732	SG	2007	
GQ357731	SG	2007	
GQ357730	SG	2007	
GQ357729	SG	2007	
GQ357728	SG	2007	
GQ357727	SG	2007	
GQ357726	SG	2007	
GQ357725	SG	2007	
GQ357716	SG	2007	
GQ357715	SG	2007	
FJ158608	CN	Aug_2007	
EU448428	VN	2007	
EU069585	SG	2004	
JN030342	SG	2010	
JN030341	SG	2010	
FJ024475	CO	Guaviare	2005
JN851129	SG	2006	
JN851127	SG	2004	
JN851118	SG	2008	
JN380849	SG	2010	
JN380848	SG	2010	
JN380843	SG	2009	
JN380822	SG	2009	
JN380820	SG	2009	
JN380818	SG	2009	
JN380813	SG	2009	
JN380812	SG	2009	
JN380811	SG	2009	
JN196592	SG	2009	
JN196591	SG	2009	
JN196590	SG	2009	
JN196586	SG	2009	
JN196572	SG	2009	
JN196571	SG	2009	
JN196570	SG	2009	
JN196569	SG	2009	
JN030305	SG	2010	
JN030296	SG	2007	
JN030269	SG	2008	
JN030242	SG	2010	
JN030241	SG	2010	
JN030240	SG	2010	
JN030239	SG	2010	
JN030218	SG	2008	
JN030217	SG	2008	
JN030212	SG	2008	
JN030211	SG	2008	
JN030205	SG	2008	
JN030204	SG	2008	
JN030203	SG	2008	
JN030202	SG	2008	

JN009092	CN	2010	
JF968032	ID	2010	
JF968025	SG	2010	
JF967957	ID	2008	
GQ357830	SG	2008	
GQ357827	SG	2007	
GQ357826	SG	2007	
GQ357823	SG	2007	
GQ357822	SG	2007	
GQ357821	SG	2007	
GQ357820	SG	2008	
GQ357817	SG	2008	
GQ357814	SG	2008	
GQ357777	SG	2007	
GQ357772	SG	2008	
GQ357766	SG	2008	
GQ357765	SG	2008	
GQ357761	SG	2008	
GQ357760	SG	2008	
GQ357758	SG	2008	
GQ357753	SG	2008	
GQ357752	SG	2008	
GQ357745	SG	2008	
EU069578	SG	2004	
JQ896296	IE	2011	
JN030285	SG	2008	
FJ024474	CO	Guaviare	2005
FJ024473	CO	Guaviare	2005
JN851115	SG	2007	
JN380850	SG	2010	
JN380823	SG	2009	
JN380821	SG	2009	
JN380819	SG	2009	
JN380817	SG	2009	
JN380815	SG	2009	
JN380814	SG	2009	
JN196609	SG	2010	
JN196600	SG	2009	
JN196599	SG	2009	
JN196573	SG	2009	
JN030320	SG	2010	
JN030312	SG	2010	
JN030308	SG	2010	
JN030304	SG	2010	
JN030303	SG	2010	
JN030302	SG	2010	
JN030292	SG	2008	
JN030268	SG	2009	
JN030261	SG	2009	
JN030260	SG	2009	
JN030237	SG	2008	
JN030236	SG	2010	
JN030235	SG	2008	
JN030233	SG	2008	
JN030230	SG	2008	
JN030215	SG	2008	

JN030214	SG	2008
JF967980	ID	2009
JF967979	ID	2009
JF967959	ID	2008
HM067747	SG	2009
HM067746	SG	2009
GQ357818	SG	2008
GQ357815	SG	2007
GQ357807	SG	2008
GQ357780	SG	2008
GQ357779	SG	2007
GQ357757	SG	2008
GQ357755	SG	2008
GQ357722	SG	2008
GQ357719	SG	2008
DQ518644	TW	1998
DQ518634	ID	2004
FJ807639	IN	1993
FJ807636	IN	2005
FJ807633	IN	2005
JN544381	SG	2010
JN544378	SG	2009
JN544357	SG	2011
JN544356	SG	2011
JN544355	SG	2011
JN544354	SG	2011
JN544352	SG	2011
JN380855	SG	2010
JN380854	SG	2010
JN380853	SG	2010
JN380852	SG	2010
JN380851	SG	2010
JN380847	SG	2010
JN380846	SG	2010
JN380840	SG	2009
JN380839	SG	2009
JN380837	SG	2009
JN380831	SG	2009
JN380829	SG	2009
JN380828	SG	2009
JN380824	SG	2009
JN196607	SG	2010
JN196606	SG	2010
JN196605	SG	2010
JN196604	SG	2010
JN196603	SG	2010
JN196602	SG	2010
JN196601	SG	2009
JN196597	SG	2010
JN196596	SG	2010
JN196580	SG	2009
JN030323	SG	2010
JN030322	SG	2010
JN030311	SG	2010
JN030310	SG	2010
JN030309	SG	2010

JN030298	SG	2010
JN030297	SG	2010
JN030295	SG	2010
JN030294	SG	2010
JN030290	SG	2009
JN030289	SG	2010
JN030288	SG	2010
JN030284	SG	2010
JN030283	SG	2010
JN030281	SG	2008
JN030279	SG	2008
JN030278	SG	2008
JN030277	SG	2008
JN030276	SG	2010
JN030275	SG	2010
JN030272	SG	2010
JN030263	SG	2010
JN030262	SG	2010
JN030231	SG	2010
JN030229	SG	2010
JN030228	SG	2010
JN030227	SG	2010
JN030226	SG	2010
JN030225	SG	2010
JN030224	SG	2010
JN030223	SG	2010
JN030222	SG	2010
JN030221	SG	2010
JN030220	SG	2010
JN030219	SG	2010
JF968049	MY	2010
JF968034	SG	2010
JF967965	MY	2008
GQ357812	SG	2008
GQ357809	SG	2008
GQ357806	SG	2008
GQ357805	SG	2008
GQ357803	SG	2008
GQ357769	SG	2008
JN851121	SG	2008
JN544380	SG	2009
JN544379	SG	2011
JN544376	SG	2011
JN544374	SG	2011
JN544373	SG	2011
JN544372	SG	2011
JN544371	SG	2011
JN544370	SG	2011
JN544359	SG	2011
JN544353	SG	2011
JN544351	SG	2011
JN544350	SG	2011
JN544349	SG	2011
JN544348	SG	2011
JN380862	SG	2010
JN380857	SG	2010

JN380856	SG	2010
JN380833	SG	2009
JN196608	SG	2010
JN196595	SG	2010
JN196593	SG	2010
JN196589	SG	2010
JN196588	SG	2010
JN196587	SG	2010
JN196585	SG	2010
JN196584	SG	2010
JN196583	SG	2010
JN196582	SG	2010
JN196581	SG	2010
JN030321	SG	2008
JN030319	SG	2010
JN030318	SG	2010
JN030313	SG	2010
JN030306	SG	2010
JN030301	SG	2010
JN030300	SG	2010
JN030299	SG	2010
JN030293	SG	2010
JN030291	SG	2010
JN030286	SG	2010
JN030282	SG	2009
JN030270	SG	2009
JN030267	SG	2010
JN030266	SG	2008
JN030265	SG	2008
JN030259	SG	2010
JN030258	SG	2010
JN030257	SG	2010
JN030256	SG	2010
JN030255	SG	2010
JN030254	SG	2010
JN030253	SG	2010
JN030252	SG	2010
JN030251	SG	2010
JN030250	SG	2010
JN030249	SG	2010
JN030248	SG	2010
JN030246	SG	2010
JN030245	SG	2008
JN030244	SG	2008
JN030243	SG	2008
JF968051	SG	2010
JF967985	ID	2009
JF967982	ID	2009
HM067745	SG	2008
GQ357813	SG	2008
GQ357811	SG	2008
GQ357808	SG	2008
GQ357802	SG	2008
GQ357713	SG	2008
GQ357712	SG	2008
FJ807634	IN	1994

JN544375	SG	2011
JN544369	SG	2011
JN544368	SG	2011
JN544367	SG	2011
JN544366	SG	2011
JN544365	SG	2011
JN544364	SG	2011
JN544363	SG	2011
JN544362	SG	2011
JN544361	SG	2011
JN544360	SG	2011
JN544358	SG	2011
JN380859	SG	2010
JN380858	SG	2010
JN380842	SG	2009
JN380836	SG	2009
JN380835	SG	2009
JN380834	SG	2009
JN380832	SG	2009
JN380827	SG	2009
JN380826	SG	2009
JN380825	SG	2009
JN196598	SG	2010
JN196594	SG	2010
JN030316	SG	2010
JN030307	SG	2010
JN030287	SG	2010
JN030280	SG	2010
JN030264	SG	2008
GQ357801	SG	2008
JN380845	SG	2009
JN030315	SG	2010
JN030314	SG	2010
JF968044	ID	2010
EU005258	GH	2005
JN030324	SG	2010
FJ807638	IN	1996
FJ538910	IN	1964
FJ538928	IN	1960
FJ538915	IN	1967
DQ448238	IN	2001
FJ538927	IN	1957
FJ538918	IN	1971
FJ538917	IN	1971
FJ538916	IN	1971
FJ538926	IN	1956
FJ538914	IN	1963
HM582106	WS	1972
FJ538921	IN	1980
HM582117	TO	1974
HM582116	TO	1974
HM582114	TO	1974
HM582113	TO	1974
HM582112	TO	1974
HM582111	TO	1974
HM582109	TA	1971

HM582108	TA	1972	
HM582107	WS	1972	
HM582105	WS	1972	
HM582104	WS	1972	
HM582100	FJ	1971	
HM582099	FJ	1971	
HM582115	TO	1974	
HM582102	NC	1971	
HM582101	FJ	1971	
GQ868590	MX	1992	
FJ538908	IN	1964	
FJ538907	IN	1964	
HM582110	TA	1973	
HM582103	NC	1972	
GQ868600	PR	1969	
GQ398257	ID	1977	
FJ538909	IN	1964	
EU056811	PE	1995	
FJ538919	IN	1971	
EU056812	PR	1977	
DQ917243	PR	1977	
DQ917242	PE	1995	
FJ807635	IN	2005	
GQ868592	CO	1986	
FJ898449	HN	1984	
FJ467493	MY	2008	
EF105379	MY	1970	
EF105387	NG	1966	
EU003591	NG	1966	
EF105385	SN	1974	
EF105388	NG	1966	
EF105386	BF	1980	
DQ917247	BF	1980	
EF105381	CI	1980	
DQ917244	CI	1980	
EF105382	BF	1980	
DQ917246	BF	1980	
HQ012533	BR	RJ	1990
HQ012538	BR	RJ	1990
HQ012534	BR	RJ	1991
HQ012510	BR	RJ	1995
HQ012511	BR	RJ	1995
HQ012512	BR	RJ	1996
HQ012516	BR	RJ	1998
HQ012536	BR	RJ	1998
HQ012517	BR	RJ	1999
HQ012537	BR	RJ	1999
HQ012518	BR	RJ	2000
HQ012519	BR	RJ	2000
HQ012520	BR	RJ	2001
HQ012521	BR	RJ	2001
HQ012522	BR	RJ	2002
HQ012525	BR	RJ	2007
HQ012526	BR	RJ	2007
HQ012527	BR	RJ	2008
HQ012528	BR	RJ	2008

HQ026763	BR	RJ	2008
HQ012531	BR	RJ	2010
HQ012532	BR	RJ	2010
GQ368158	BR	RJ	1998
GQ368159	BR	RJ	1998
GQ368160	BR	RJ	1998
GQ368173	BR	RJ	1998
GQ368174	BR	RJ	1998
GQ368161	BR	RJ	2007
GQ368162	BR	RJ	2007
GQ368163	BR	RJ	2007
GQ368164	BR	RJ	2007
GQ368165	BR	RJ	2008
GQ368166	BR	RJ	2008
GQ368167	BR	RJ	2008
GQ368168	BR	RJ	2008
GQ368169	BR	RJ	2008
GQ368170	BR	RJ	2008
GQ368171	BR	RJ	2008
GQ368172	BR	RJ	2008
GQ368175	BR	RJ	2008
GQ368176	BR	RJ	2008
JQ710658	BR	RJ	2010
JQ710657	BR	RJ	2011
HQ012523	BR	ES	2002
HQ012524	BR	ES	2003
HQ012530	BR	ES	2009
GQ868549	BR	SP	2008
GQ868550	BR	SP	2008
GQ868551	BR	SP	2008
GU131864	BR	SP	2008
GU131879	BR	SP	2008
GU131880	BR	SP	2008
GU131881	BR	SP	2008
GU131882	BR	SP	2008
GU131883	BR	SP	2008
GU131884	BR	SP	2008
GU131885	BR	SP	2008
HM181971	BR	SP	2008
GQ330472	BR	SP	2009
HQ012508	BR	BA	1991
HQ012509	BR	CE	1994
HQ012535	BR	CE	1994
HQ012513	BR	BA	1996
HQ012514	BR	RN	1997
HQ012515	BR	RN	1997
HQ012529	BR	BA	2009
JN819419	BR	Northern	2000
FJ850072	BR	Northern	2000
FJ850074	BR	Northern	2001
FJ850076	BR	Northern	2002
FJ850078	BR	Northern	2003
GQ868640	BR	Northern	2003
FJ850082	BR	Northern	2004
FJ850085	BR	Northern	2005
FJ850088	BR	Northern	2006

FJ850091	BR	Northern	2007
GQ199890	BR	Northern	2008
AY484622	AN	Curacao	1996
AY484658	AN	Curacao	1993
AY484619	AW	1996	
AY484648	BB	1987	
AY484620	BB	1998	
AY484650	BB	1988	
AY484638	BS	1998	
AY484624	DM	1995	
AY484627	DM	1995	
AY484656	DM	1995	
AY484625	DO	1984	
AY484626	DO	1990	
AY484629	GD	1999	
AY484616	LC	1999	
AY484617	LC	1999	
AY484621	LC	1999	
AY484631	LC	1999	
AY484666	SV	1987	
AY484615	TT	1997	
AY484618	TT	1997	
AY484632	TT	2000	
AY484633	TT	1997	
AY484639	TT	1986	
AY484640	TT	1987	
AY484641	TT	1988	
AY484643	TT	1986	
AY484645	TT	1989	
AY484646	TT	1996	
AY484647	TT	1986	
AY484653	TT	2000	
AY484657	TT	1996	
AY484659	TT	1986	
AY484667	TT	1981	
AY484623	VC	1998	
AY484628	VC	1998	
AY484630	VC	1998	
AY484636	VC	1998	
AY484613	SR	1993	
AY484612	SR	1999	
AY484610	SR	1993	
AY484609	SR	1993	
AY484614	SR	1999	
AY484634	SR	1986	
AY484635	SR	1986	
AY484637	SR	1986	
AY484660	SR	1999	
AY484654	SR	1993	
AY484652	SR	1986	
AY484642	GY	2000	
AY484611	BO	1997	
AY484644	CO	1993	
AY484655	VE	1990	
AY484651	EC	2000	
AY484649	EC	2000	

AY484661	HN	1986
AY484662	HN	1991
AY484663	HN	1991
AY484664	HN	1991
AY484665	HN	1991
AY484605	JM	1983
AY702041	CU	1997
AY702042	CU	1997
AY702043	CU	1997
AY702044	CU	1997
AY702045	CU	1997
AY702046	CU	1997
AY702047	CU	1997
AY702048	CU	1997
AY702049	CU	1997
AY702050	CU	1997
AY702051	CU	1997
AY702052	CU	1997
AY702053	CU	1997
AY702054	CU	1997
AY702055	CU	1997
AY702056	CU	1997
AY702057	CU	1997
AY702058	CU	1997
AY702059	CU	1997
AY702060	CU	1997
JX051785	PE	2010
JX051792	PE	2010
JX051791	PE	2010
JX051790	PE	2010
JX051788	PE	2010
JX051789	PE	2010
JX051787	PE	2010
JX051796	PE	2011
JX051786	PE	2010
JX051793	PE	2011
KC847996	PE	2012
JX051795	PE	2011
JX051794	PE	2011
KC847995	PE	2012
KC847991	PE	2011
KC847994	PE	2012
JX051784	PE	2009
JX051783	PE	2009
KC847992	PE	2012
KC847993	PE	2012
JX051797	PE	2011
JX051798	PE	2011
JX051777	PE	2007
JX051778	PE	2007
JX051779	PE	2007
JX051780	PE	2008
JX051782	PE	2009
JX051781	PE	2009
JX051772	PE	2002
JX051771	PE	2002

JX051773	PE	2002
JX051774	PE	2002
JX051775	PE	2002
JX051776	PE	2002
JX051769	PE	2001
JX051770	PE	2001
JX051767	PE	2000
JX051768	PE	2000
AF100466	VE	1990
AY158329	VE	1990
AF398106	VE	1999
AF363078	VE	2008
AF363092	VE	2008
AF398108	VE	1999
AF398113	VE	2000
FJ182012	CO	2005
JX051806	BO	2007
JX051801	BO	2003
JX051802	BO	2007
JX051804	BO	2007
JX051805	BO	2007
JX051808	BO	2006
JX051809	BO	2007
JX051803	BO	2007
JX051807	BO	2010
JX051810	BO	2010
JX051811	BO	2010
JX051812	PY	2010
JX051814	PY	2010
JX051813	PY	2010
