# Supplementary material for: Spatiotemporal Dynamics of DENV-2 Asian-American Genotype Lineages in the Americas
Source: PLoS One. 2014 Jun 4;9(6):e98519. doi: 10.1371/journal.pone.0098519 (PMC4045713; doi:10.1371/journal.pone.0098519)
Supplement: Table S2 — Posterior state probability distributions for the roots of major DENV-2 American lineages estimated using two different schemes of grouping of discrete geographic states. (PDF) [file pone.0098519.s002.pdf]

Table S2. Posterior state probability distributions for the roots of major DENV-2 American lineages estimated using two different schemes of grouping of discrete geographic states.

Group scheme	MRCA	Posterior State Probability					
		GA	LA/SR/GY	SA1*	SA2*	CAM*	PE
6 geographic states	DENV-2 AS/AM (America)	0.71	0.29	0	0	0	0
	Lineage I	0	1	0	0	0	0
	Lineage II	1	0	0	0	0	0
	Lineage III	0.39	0.61	0	0	0	0
	Lineage IV	0.99	0.01	0	0	0	0
10 geographic states	DENV-2 AS/AM (America)	0.69	0.31	0	0	0	0
	Lineage I	0	1	0	0	0	0
	Lineage II	1	0	0	0	0	0
	Lineage III	0.36	0.63	0	0.01	0	0
	Lineage IV	0.98	0.02	0	0	0	0

For the analysis with 10 locations, the SA1 location was split in three (BR-SE, BR-N/NE and BO/PY), the SA2 location was split in two (VE and CO/EC), and the CAM location was split in two (NI and CAM/MX).
