# Supplementary material for: Spatiotemporal Dynamics of DENV-2 Asian-American Genotype Lineages in the Americas
Source: PLoS One. 2014 Jun 4;9(6):e98519. doi: 10.1371/journal.pone.0098519 (PMC4045713; doi:10.1371/journal.pone.0098519)
Supplement: Table S3 — Viral migration rates between locations. (PDF) [file pone.0098519.s003.pdf]

Table S3. Viral migration rates between locations.

From	To					
	GA	LA/SR/GY	SA-1	SA-2	PE	CEN
GA	-	7.26	1.14	1.52	0.03	0.21
LA/SR/GY	3.92	-	1.41	2.18	0.05	0.75
SA-1	0.08	0.07	-	0.08	4.13	0.03
SA-2	0.86	0.10	0.80	-	2.68	1.12
PE	0.05	0.02	0.13	0.24	-	0.02
CEN	0.06	0.05	0.03	1.11	0.04	-
